# Antioxidant enzyme and DNA base repair genetic risk scores’ associations with systemic oxidative stress biomarker in pooled cross-sectional studies

**DOI:** 10.3389/fragi.2023.1000166

**Published:** 2023-04-21

**Authors:** Ziling Mao, Abigail L. H. Gray, Bharat Thyagarajan, Roberd M. Bostick

**Affiliations:** ^1^ Department of Epidemiology, Rollins School of Public Health, Emory University, Atlanta, GA, United States; ^2^ Department of Laboratory Medicine and Pathology, University of Minnesota, Minneapolis, MN, United States; ^3^ Winship Cancer Institute, Emory University, Atlanta, GA, United States

**Keywords:** oxidative stress, oxidative balance, F2-isoprostanes, genetic risk score, antioxidant enzymes, DNA base excision repair, oxidative balance score, cross-sectional studies

## Abstract

**Background:** Oxidative stress is hypothesized to contribute to the pathogenesis of several chronic diseases. Numerous dietary and lifestyle factors are associated with oxidative stress; however, little is known about associations of genetic factors, individually or jointly with dietary and lifestyle factors, with oxidative stress in humans.

**Methods:** We genotyped 22 haplotype-tagging single nucleotide polymorphisms (SNPs) in 3 antioxidant enzyme (AE) genes and 79 SNPs in 14 DNA base excision repair (BER) genes to develop oxidative stress-specific AE and BER genetic risk scores (GRS) in two pooled cross-sectional studies (*n* = 245) of 30–74-year-old, White, cancer- and inflammatory bowel disease-free adults. Of the genotypes, based on their associations with a systemic oxidative stress biomarker, plasma F_2_-isoprostanes (FiP) concentrations, we selected 4 *GSTP1* SNPs for an AE GRS, and 12 SNPs of 5 genes (*XRCC1*, *TDG*, *PNKP*, *MUTYH*, and *FEN1*) for a BER GRS. We also calculated a previously-reported, validated, questionnaire-based, oxidative stress biomarker-weighted oxidative balance score (OBS) comprising 17 anti- and pro-oxidant dietary and lifestyle exposures, with higher scores representing a higher predominance of antioxidant exposures. We used general linear regression to assess adjusted mean FiP concentrations across GRS and OBS tertiles, separately and jointly.

**Results:** The adjusted mean FiP concentrations among those in the highest relative to the lowest oxidative stress-specific AE and BER GRS tertiles were, proportionately, 11.8% (*p* = 0.12) and 21.2% (*p* = 0.002) higher, respectively. In the joint AE/BER GRS analysis, the highest estimated mean FiP concentration was among those with jointly high AE/BER GRS. Mean FiP concentrations across OBS tertiles were similar across AE and BER GRS strata.

**Conclusion:** Our pilot study findings suggest that DNA BER, and possibly AE, genotypes collectively may be associated with systemic oxidative stress in humans, and support further research in larger, general populations.

## 1 Introduction

Oxidative stress, which has been defined as an unfavorable imbalance of pro- oxidants relative to anti- oxidants (Sies, 1997), has been implicated in the pathogenesis of several chronic diseases (Salminen et al., 2012; Schottker et al., 2015a; Schottker et al., 2015b). Multiple dietary and lifestyle factors, as well as genetic variation, likely contribute to systemic oxidative stress (Storz et al., 1990; Kelishadi et al., 2009; Vetrani et al., 2013). Reactive oxygen and nitrogen species (RONS) are oxidants that are formed normally during aerobic metabolism, and are kept in balance by antioxidants, which delay or inhibit oxidation (Sies, 1997). Excess RONS production leads to cell and DNA damage (Storz and Imlayt, 1999; Sies, 2000). Since antioxidant enzymes (AEs) and the DNA base excision repair (BER) pathway contribute to reducing RONS or mitigating RONS-induced damage (Sun, 1990; Salmon et al., 2004; Rowe et al., 2008; Maynard et al., 2009), AE and BER genes may play important roles in oxidative stress regulation, and polymorphisms in these genes may be related to variability in that regulation.

For the present study, we measured plasma F_2_-isoprostanes (FiP) concentrations, one of the most reliably-measured, widely-used systemic oxidative stress biomarkers in epidemiologic studies (Morrow and Roberts, 1997; Liu et al., 1999; Roberts and Morrow, 2000; Montuschi et al., 2004; Milne et al., 2005). We recently developed and validated a novel, questionnaire-based, oxidative stress biomarker-weighted oxidative balance score (OBS) to reflect the combined contributions of multiple dietary and lifestyle exposures to systemic oxidative balance (Mao and Bostick, 2022). The rationale for creating a comprehensive score that incorporates multiple dietary nutrients and lifestyle exposures was previously described (Dash et al., 2013; Dash et al., 2015; Mao et al., 2020). The novel OBS was weighted based on the strength of association of each score component with plasma FiP concentrations in two pooled cross-sectional studies (included participants in the present study; further details provided in the Methods section) (Mao and Bostick, 2022). The novel, oxidative stress biomarker-weighted OBS was associated with plasma FiP concentrations more strongly than was an equal-weight OBS (Mao and Bostick, 2022).

Most individual dietary and lifestyle factors have tended to be modestly and inconsistently associated with oxidative stress, but in combination have been strongly and consistently associated with it (Byrd et al., 2019); similarly, it would seem unlikely that relatively common single AE or BER gene polymorphisms would be strongly associated with oxidative stress, but in aggregate they might. Genetic risk scores (GRS) that combine multiple variants in multiple genes have been associated with chronic disease outcomes (Meigs et al., 2008; Conran et al., 2016), and a GRS based on susceptibility variants for elevated fasting glucose concentrations was associated with oxidative stress biomarkers (Kim et al., 2018). However, we found no reported associations of AE, BER, or other GRS, alone or in interaction with dietary and lifestyle exposures, with oxidative stress biomarkers.

Accordingly, we report a pilot study to develop oxidative stress-specific AE and BER GRS and estimate their associations with circulating FiP concentrations, individually and jointly with a novel, oxidative stress biomarker-weighted OBS in two pooled cross-sectional studies. We hypothesized that 1) higher GRS would be associated with higher FiP concentrations (our primary hypothesis), 2) the OBS would be associated with lower FiP concentrations (a secondary hypothesis since the analysis to address it was an update of one in our previous report of an inverse OBS-FiP association (Mao and Bostick, 2022)), 3) the inverse OBS-FiP association would be strongest among those who also had a high AE or BER GRS (exploratory hypotheses), and 4) the two GRS would interact such that those who had both a high AE and a high BER GRS would have the highest FiP concentration of any AE/BER GRS combination (an exploratory hypothesis).

## 2 Materials and methods

### 2.1 Study population

We pooled data from two, methodologically nearly identical, cross-sectional studies of patients going for outpatient, elective colonoscopies: the Markers of Adenomatous Polyps (MAP) I study (1994—1997), conducted in Winston-Salem and Charlotte, North Carolina, and the MAP II study (2002), conducted in Columbia, South Carolina. Details of the two studies were reported previously (Boyapati et al., 2003; Daniel et al., 2009). Briefly, participants were recruited from patients without a prior history of colorectal neoplasms who were scheduled for outpatient, elective colonoscopies at large gastroenterology clinics. Eligibility included being 30—74 years of age, in general good health, capable of informed consent, and English-speaking; exclusions included a history of colorectal adenomatous polyps, cancer (other than non-melanoma skin cancer), inflammatory bowel disease, known genetic syndromes associated with colonic neoplasia, liver disease, and others. The consent rates for the MAP I and MAP II studies were, respectively, 67% and 76%; the sample sizes were, respectively, 526 and 267 (yielding an initial pooled sample size of 793).

Each study was approved by the Institutional Review Board (IRB) of the institution where it was conducted: Wake Forest University School of Medicine for MAP I and the University of South Carolina for MAP II. All participants provided written informed consent. The present analysis, which was conducted at Emory University as a secondary analysis using de-identified data, was exempt from further IRB review. Hereinafter, the MAP I and the MAP II studies are referred to as the pooled MAP studies.

### 2.2 Data collection

Prior to the colonoscopy visit, all study participants completed mailed questionnaires concerning demographics, medical history, family history of colorectal cancer, reproductive history (in women), anthropometrics (self-reported), lifestyle, and diet. Diet was assessed with semi-quantitative Willett food frequency questionnaires (FFQ) (Willett et al., 1988), and physical activity with modified Paffenbarger questionnaires (Paffenbarger et al., 1993), which queried usual lengths of times spent in specified moderate and vigorous activities on weekdays and weekends. We summed the durations spent in each physical activity category, calculated the metabolic equivalents of task (METs) hours/week, and then summed the MET-hours/week from moderate and vigorous physical activities. We calculated body mass index (BMI) as weight (kg) divided by height squared (m^2^), and a waist-to-hip ratio, as waist circumference divided by hip circumference.

Participants completed questionnaires at home, and at their colonoscopy visit submitted them and had fasting venous blood samples taken prior to their colonoscopy procedure. Blood was collected, handled, and stored so as to allow genotyping and biomarker measurements. The fasting venous blood samples were drawn into pre-chilled, red-coated Vacutainer tubes, which were immediately placed on ice and shielded from light, and taken to the laboratory, where the tubes for serum and plasma were immediately centrifuged under refrigeration, and aliquoted into amber-colored cryopreservation vials. Butylated hydroxytoluene and salicylic acid, lipid and aqueous soluble antioxidants, respectively, were added to aliquots designated for oxidative stress biomarker measurements. The air in all aliquot vials was displaced with inert gas (nitrogen in MAP I and argon in MAP II), then the vials were capped with O-ring screw caps. The aliquots were then immediately placed in −70°C freezers until analysis. The FiP assays were conducted at the University of Minnesota’s Molecular Epidemiology and Biomarker Research Laboratory. FiP concentrations were assayed using a gas chromatography-mass spectrometry method (Morrow and Roberts, 1994; Milne et al., 2007) considered the gold standard method for measuring FiP. FiP was extracted from participants’ samples using deuterium (4)-labeled 8-iso-prostaglandin F_2_α as an internal standard. Quality control procedures included analysis of two control pools with varying FiP concentration ranges; the inter-assay coefficients of variation were 9.5% and 11%.

We used a multistep process to select genes and single nucleotide polymorphisms (SNPs) for possible inclusion in genetic risk scores for two pathways—antioxidant enzymes (AEs) and DNA base excision repair (BER). We used NIEHS SNPs and Seattle SNPs to identify AE and BER genes known to have haplotype-tagging SNPs (tagSNPs) and/or functional SNPs at the time the study was conducted. Functional SNPs were identified using bioinformatic algorithms such as SIFT (Sorting Intolerant from Tolerant) (Ng and Henikoff, 2002) and PolyPhen (Ramensky et al., 2002). Non-synonymous SNPs that were identified by one or both of these algorithms to impact either evolutionary conservation or protein function and having a minor allele frequency (MAF) > 3.5% were considered functional SNPs. Then, using the Phase II HapMap CEPH population, we selected tagSNPs with a MAF cutoff of >5% and a *r* ^2^ threshold of 0.80. Thus, for the AE genes, we selected for genotyping 6 SNPs for *MnSOD*, 5 for *GTSP1*, and 11 for *CAT* (see Supplementary Table S1); and for the BER pathway genes, we selected for genotyping 11 SNPs for *XRCC1*, 2 for *UNG*, 11 for *TDG*, 4 for *SMUG1*, 3 for *POLB*, 3 for *PNKP*, 6 for *OGG1*, 6 for *MUTYH*, 3 for *MPG*, 6 for *MBD4*, 5 for *LIG3*, 15 for *LIG1*, 1 for FEN1, and 3 for *APEX1* (see Supplementary Table S2). Genotyping was conducted at the Biomedical Genomics Center, the core genotyping laboratory at the University of Minnesota, using the iPLEX Sequenom genotyping platform. For 64 pairs of blinded duplicate samples, the genotyping concordance for the selected SNPs was ≥95% (Wang et al., 2017).

### 2.3 Data analyses

#### 2.3.1 Exclusion criteria

We excluded from analysis 292 participants missing plasma FiP concentration values (due to insufficient remaining plasma), 2 with extreme (>380 pg/ml) FiP values, 184 missing genotyping on more than 20% of the selected AE and BER genes, 30 non-White participants (because of population stratification and insufficient sample size for separate genetic analyses), 12 missing data on lifestyle factors, 10 missing >15% of their FFQ responses, 10 who reported implausible total energy intakes (<600 or >6,000 kcal/d), and 8 with extreme (>10,000 IU/day) supplemental carotene intakes. Thus, the final sample size for analysis was 245 (151 from MAP I, and 94 from MAP II).

#### 2.3.2 Oxidative balance score (OBS)

We originally developed and validated the oxidative stress biomarker-weighted, 17-component OBS to represent the aggregate contributions of dietary nutrients and lifestyle characteristics to oxidative stress in a larger (*n* = 386) pooled MAP studies population, which included participants who were not White and/or were missing genotyping data (Mao and Bostick, 2022). Briefly, as previously reported, all score components were chosen *a priori* based on their literature-supported physiological anti- or pro-oxidant effects (Dash et al., 2013; Dash et al., 2015; Mao et al., 2020), and included dietary and supplemental antioxidants (vitamins C and E, α and β carotene, lutein, lycopene, flavonoids, glucosinolates, omega-3 fatty acids, selenium, and calcium), dietary pro-oxidants (iron, omega-6 fatty acids, and saturated fats), and lifestyle factors, including alcohol intake, BMI, smoking (all three considered to have predominantly pro-oxidant effects), and physical activity (considered to have predominantly antioxidant effects).

As previously reported (Mao and Bostick, 2022), we calculated weights for each OBS component in the pooled MAP studies based on the strength of its multivariable-adjusted, individual association with plasma FiP concentrations. To do this, we first standardized each dietary component (all continuous) by study and sex, to a mean of 0 and standard deviation (SD) of 1.0. For lifestyle exposures, we created dummy variables since all components were categorical variables. Next, we transformed the FiP values by the natural logarithm to meet normality assumptions, ruled out multicollinearity, and then used multivariable linear regression to estimate the maximum likelihood estimates for β-coefficients, which represent the average change in plasma FiP concentrations per 1 standard deviation increase in a dietary component or having a certain lifestyle behavior relative to its referent category. Next, we assigned weights to each component equal to 10 times its corresponding β-coefficient from the multivariable linear regression models. Finally, we added the components’ values to yield a participant’s OBS, such that a higher score would represent a higher balance of anti- to pro-oxidant exposures (as we previously reported, a higher balance was associated with lower systemic oxidative stress as indicated by lower circulating FiP concentrations).

#### 2.3.3 Genetic risk scores (GRS)

We used a multistep process to develop the oxidative stress-specific AE and BER GRS from the genotyped SNPs identified in Section 2.2. First, we tested all SNPs for Hardy-Weinberg equilibrium, then combined variant allele heterozygotes and homozygotes when ≤10 participants had either genotype. Then, for each genotype, we calculated and compared mean plasma FiP concentrations using sex- and BMI-adjusted general linear models. Then, from these results, we calculated proportional mean differences in FiP concentrations between the variant genotypes and the common homozygote as: (variant genotype—common homozygote)/common homozygote x 100% (Supplementary Tables S3, S4). For this pilot investigation, we selected SNPs for inclusion in the two GRS based on considerations of the estimated magnitudes of the proportional mean FiP differences and the variability of the estimates, as reflected by the *p*-values for the estimated magnitudes. For the AE GRS, we included SNPs if the proportional mean differences in FiP concentrations were >5% with a *p*-value ≤0.05, or if the proportional mean differences in FiP concentrations were >10% with a *p*-value ≤0.15. Similarly, we included SNPs in the BER GRS if the proportional mean differences in FiP concentrations were >10% with a *p*-value ≤0.15. Based on these SNP selection criteria, for the oxidative stress-specific AE GRS we included 4 *GSTP1* SNPs (Supplementary Table S5); and for the oxidative stress-specific BER GRS we included, 2 SNPs for *XRCC*, 2 for *TDG*, 5 for *PNKP*, 2 for *MUTYH* and 1 for *FEN1* (Supplementary Table S6).

Next, for each SNP we included in a GRS, we assigned each variant allele 1 point and gave it a positive sign if the mean biomarker concentration was higher among those with the variant allele, and a negative sign if it was lower. Finally, we summed the values assigned to the genotypes to yield the respective GRS such that a higher GRS would indicate a higher balance of variant alleles directly associated with FiP relative to variant alleles inversely associated with FiP (thus, a higher balance was hypothesized to be associated with higher systemic oxidative stress as indicated by higher circulating FiP concentrations).

#### 2.3.4 Statistical analyses

We summarized and compared the selected characteristics of the study participants across FiP concentration tertiles using chi-square tests for categorical variables and general linear models for continuous variables (transformed by the natural logarithm to meet normality assumptions when indicated).

We calculated and compared adjusted mean FiP concentrations across categories (tertiles for the BER GRS, and dichotomized categories for the AE GRS due to the small number of SNPs included) of the GRS and tertiles of the OBS using multivariable general linear models. In addition, to assess potential modification of the OBS-FiP associations by the two GRS, we further assessed the associations of the OBS with FiP concentrations stratified by dichotomized AE and BER GRS. Since we log transformed FiP concentrations, we calculated and report geometric means and their 95% confidence intervals (CI). For the GRS models we *a priori* included sex and the OBS as covariates. For the OBS models, we selected the covariates based on a combination of previous research, biologic plausibility, and whether inclusion/exclusion of potential covariates from the model affected the estimated proportional difference in the mean concentration of the biomarker of interest between the highest and lowest OBS tertile by 10% or more. The covariates selected for the final OBS model included total energy intake, sex, current hormone replacement therapy (HRT) use, education, and regular (≥once/week) non-steroidal anti-inflammatory drug (NSAID) use.

Finally, we assessed potential interaction between the oxidative stress-specific AE and BER GRS in relation to adjusted mean FiP concentrations, *via* a joint/combined analysis in which participants in the joint lowest AE GRS category/BER GRS category were the reference group.

We used SAS version 9.4 software (SAS Institute, Inc. Cary, North Carolina) to conduct all statistical analyses, and considered two-sided *p*-values ≤0.05 statistically significant.

## 3 Results

We summarize selected characteristics of the participants according to plasma FiP concentration tertiles in Table 1. Among the 245 participants on whom FiP was measured, the plasma FiP concentration range was 29.32—223.43 pg/ml. Participants in the highest relative to the lowest FiP tertile were more likely to be female and currently smoke; less likely to have a college degree or higher, a high alcohol intake, or take aspirin regularly; and, on average, to have a higher BMI and total vitamin C and E intakes, and a lower OBS and circulating serum 25-OH-vitamin D_3_ concentrations.

**TABLE 1:** Selected characteristics of participants (*n* = 245)^a^, by tertiles of plasma F_2_-isoprostanes concentrations, in the pooled MAP I and MAP II cross-sectional studies^b^.

	Plasma F_2_-isoprostanes tertiles			
Characteristics	1	2	3	*p* ^c^
	29.32-64.57 pg/ml	64.58-94.36 pg/ml	94.37-223.43 pg/ml	
	(*n* = 81)	(*n* = 82)	(*n* = 82)	
**Demographics**				
Age (years)	57.8 ± 8.1	58.4 ± 8.1	55.5 ± 9.3	0.08
Male (%)	63.0	53.0	21.0	< 0.001
College degree or higher (%)	45.7	27.7	14.8	< 0.001
Family history of CRC in a first degree relative (%)	25.9	22.9	28.4	0.72
**Lifestyle**				
Regular^d^ NSAID use (%)	23.8	27.7	31.3	0.57
Regular^d^ aspirin use (%)	47.5	37.4	19.8	< 0.001
HRT use in women (*n* = 133) (%)	53.3	56.4	57.8	0.92
Current smoker (%)	5.7	13.9	21.7	0.08
Body mass index (kg/m^2^)	26.2 ± 4.6	27.2 ± 5.2	30.2 ± 7.9	< 0.001
Waist:hip ratio	0.925 ± 0.103	0.907 ± 0.111	0.890 ± 0.103	0.12
Heavy alcohol intake^e^ (%)	9.9	12.1	6.2	0.03
Physical activity (METs/wk)^f^	308 ± 192	328 ± 221	337 ± 201	0.64
**Serum 25-OH-vitamin D**_**3**_ **concentrations** (ng/ml)	26.3 ± 9.9	28.9 ± 13.2	22.7 ± 8.2	0.008
**Dietary intakes**				
Total energy (kcal/d)	1,944 ± 672	1,801 ± 752	1,841 ± 743	0.06
Total fat (% kcal/d)	30.3 ± 6.8	33.2 ± 7.4	31.9 ± 7.2	0.04
Total^g^ calcium (mg/1,000 kcal/d)	534 ± 331	472 ± 328	477 ± 308	0.14
Dietary fiber (g/1,000 kcal/d)	11.6 ± 3.6	10.5 ± 3.6	10.8 ± 3.4	0.39
Red and processed meats (servings/wk)	7.2 ± 6.1	7.4 ± 5.4	6.8 ± 4.8	0.47
Total fruits and vegetables (servings/wk)	41.0 ± 24.0	30.2 ± 17.6	39.2 ± 28.9	0.003
*Antioxidants*				
Total^g^ carotene (IU/1,000 kcal/d)	5,157 ± 3,636	3,391 ± 3,037	4,785 ± 4,089	0.11
Total lutein (mg/1,000 kcal/d)	1,700 ± 1,251	1,644 ± 1,435	1,558 ± 1,135	0.78
Total lycopene (mg/1,000 kcal/d)	3,045 ± 3,573	2,238 ± 1,494	3,021 ± 2,457	0.08
Total^g^ vitamin C (mg/1,000 kcal/d)	64 ± 139	53 ± 132	49 ± 138	0.07
Total^g^ vitamin E (mg/1,000 kcal/d)	56 ± 96	39 ± 96	17 ± 42	< 0.001
Total^g^ omega-3 fatty acid (g/1,000 kcal/d)	0.12 ± 0.08	0.13 ± 0.11	0.10 ± 0.10	0.37
Dietary flavonoids (mg/1,000 kcal/d)	267 ± 240	242 ± 257	265 ± 271	0.35
Dietary glucosinolates (mg/1,000 kcal/d)	9.0 ± 6.7	11.4 ± 11.5	11.5 ± 14.6	0.68
Supplemental selenium (mcg/1,000 kcal/d)	6.6 ± 16.4	5.3 ± 21.8	2.0 ± 7.9	0.76
*Prooxidants*				
Total^g^ iron (mg/1,000 kcal/d)	14.5 ± 15.3	10.6 ± 8.4	11.6 ± 16.8	0.18
Dietary omega-6 fatty acids (g/1,000 kcal/d)	6.2 ± 2.1	6.7 ± 2.8	6.2 ± 1.9	0.27
Saturated fats (g/1,000 kcal/d)	10.9 ± 2.8	12.0 ± 3.0	11.8 ± 3.0	0.02
**Oxidative balance score (OBS)** ^h^	0.35 ± 1.60	-0.58 ± 1.65	-1.51 ± 1.67	< 0.001

Abbreviations: CRC, colorectal cancer; d, day; HRT, hormone replacement therapy; MAP, markers of adenomatous polyps; MET, metabolic equivalents of task; NSAID, non-steroidal anti-inflammatory drug; wk, week.

^a^ *n* = 243 for age and NSAID, use, *n* = 244 for regular aspirin use, *n* = 188 for serum 25-OH-vitamin D_3_, and *n* = 196 for dietary flavonoids and glucosinolates due to missing data.

^b^ Data presented as mean ± standard deviation unless otherwise specified.

^c^ *p*-values based on chi-square test for categorical variables and ANOVA, for continuous variables (transformed by the natural logarithm to meet normality assumptions, when indicated).

^d^ ≥ once per week.

^e^ Heavy alcohol intake defined as > 1 drink/day for women and >2 drinks/day for men.

^f^ Moderate + vigorous physical activity.

^g^ Total intake = dietary + supplemental.

^h^ Oxidative balance score a composite of 17 weighted anti- and pro-oxidant dietary and lifestyle exposures (see text); score range −5.25—3.98; a higher score represents higher anti-relative to pro-oxidant exposures.

We summarize mean plasma FiP concentrations according to oxidative stress-specific AE and BER GRS tertiles in Table 2. In the multivariable adjusted analyses, we observed a statistically significant, dose-response pattern of increasing mean FiP concentrations across the BER GRS tertiles; the mean FiP concentration among participants in the highest relative to the lowest BER GRS tertile was, proportionately, 21.2% higher (*p* = 0.002). A similar but more modest increasing pattern was observed across the AE GRS tertiles; the mean FiP concentration among those in the highest relative to the lowest AE GRS tertile was estimated to be proportionately 11.8% higher (*p* = 0.12).

**TABLE 2:** Mean^a^ plasma F_2_-isoprostanes concentrations according to tertiles of DNA base excision repair (BER) and antioxidant enzyme (AE) genetic risk scores (GRS), in the pooled MAP I and MAP II cross-sectional studies.

GRS, model, and tertiles	GRS tertile medians	Plasma F_2_-isoprostanes, pg/mL				
		*n*	Means	(95% CI)	Prop. Diff.^b^ (%)	*p*
**BER GRS** ^c^						
Crude^d^						
1	-2	76	69.1	(63.3, 75.5)	Ref.	
2	1	101	82.2	(76.2, 88.8)	18.9	
3	4	68	92.0	(83.9, 101.0)	33.1	< 0.001
Fully-adjusted^e^						
1	-2	76	71.8	(66.6, 77.5)	Ref.	
2	1	101	83.0	(77.8, 88.6)	15.6	
3	4	68	87.0	(80.3, 94.3)	21.2	0.002
**AE GRS** ^f^						
Crude^d^						
1	-1	104	75.5	(69.9, 81.6)	Ref.	
2	2	82	85.9	(78.7, 93.7)	13.8	
3	4	59	81.9	(73.9, 90.8)	8.6	0.09
Fully-adjusted^e^						
1	-1	104	76.8	(72.0, 82.0)	Ref.	
2	2	82	81.2	(75.4, 87.5)	5.7	
3	4	59	85.9	(78.7, 93.7)	11.8	0.12

Abbreviations: AE, antioxidant enzyme; BER, base excision repair; CI, confidence interval; GRS, genetic risk score; MAP, markers of adenomatous polyps, Prop. Diff., proportional difference, Ref., reference.

^a^ Geometric means, 95% confidence intervals, and *p*-values from general linear models; unequal sample sizes in tertiles due to ranking ties; differences in the numbers of participants due to availability of blood samples for assays.

^b^ Proportional difference calculated as (comparison group mean - reference group mean)/(reference group mean) x 100%.

^c^ BER, genetic risk score based on 12 SNPs, in 5 BER, genes; see complete list of genes and SNPs, in the text and Supplementary Table S4; a higher GRS indicates a higher number of higher relative to lower risk alleles.

^d^ No covariates in the model.

^e^ Adjusted for sex and the oxidative balance score (OBS).

^f^ AE, gene score based on 4 SNPs, in the *GSTP1* gene; see complete list of SNPs, in the text and Supplementary Table S3; a higher GRS indicates a higher number of higher relative to lower risk alleles.

We previously reported the OBS-FiP association in the larger (*n* = 386) pooled MAP studies population; among those in the highest relative to the lowest OBS tertile, the multivariable-adjusted mean plasma FiP concentration was, proportionately, 29.7% lower (*p* < 0.001) (Mao and Bostick, 2022). In the present, more restricted pooled MAP studies population, the multivariable-adjusted mean FiP concentration among those in the highest relative to the lowest OBS tertile was, proportionately, 32.0% lower (*p* < 0.001; Supplementary Table S7).

We summarize multivariable-adjusted mean plasma FiP concentrations across OBS tertiles, stratified by dichotomized oxidative stress-specific BER and AE GRS in Table 3. We found no strong or statistically significant evidence for effect modification of the OBS-FiP association by either GRS. However, we noted some suggestions that the inverse OBS-FiP association was slightly stronger among those with a high oxidative stress-specific AE or BER GRS. Among those with a high oxidative stress-specific AE or BER GRS, the mean FiP concentrations among those in the highest relative to the lowest OBS tertile were estimated to be 34.3% and 31.6% lower, respectively, whereas among those with a low oxidative stress-specific AE GRS they were estimated to be 23.2% and 25.1% lower, respectively.

**TABLE 3:** Mean^a^ plasma F_2_-isoprostanes concentrations across tertiles of an oxidative balance score (OBS), stratified by dichotomized BER and AE genetic risk scores (GRS), in the pooled MAP I and MAP II cross-sectional studies.

GRS strata/OBS^b^ tertiles	OBS tertile medians	Plasma F_2_-isoprostanes, pg/mL				
		*n* ^c^	Means	(95% CI)	Prop. Diff.^d^ (%)	*p*
BER GRS^e^						
Low (<1)						
1	-3.09	48	85.9	(73.5, 100.4)	Ref.	
2	0.03	48	76.4	(66.4, 88.0)	-11.1	
3	3.74	48	64.4	(56.8, 73.0)	-25.1	0.002
High (≥1)						
1	-3.26	65	109.5	(97.6, 122.8)	Ref.	
2	0.56	65	92.6	(83.0, 103.4)	-15.4	
3	3.81	65	74.9	(66.4, 84.3)	-31.6	< 0.001
*P*_*interaction*_ ^f^						0.44
AE GRS^g^						
Low (<2)						
1	-2.46	35	94.2	(81.3, 109.0)	Ref.	
2	1.49	38	79.2	(69.1, 90.7)	-15.9	
3	3.32	38	72.3	(62.9, 83.1)	-23.2	0.003
High (≥2)						
1	-3.46	40	109.5	(96.5, 124.4)	Ref.	
2	-1.33	50	88.0	(77.9, 99.5)	-19.6	
3	3.85	44	72.0	(63.8, 81.3)	-34.3	< 0.001
*P*_*interaction*_ ^f^						0.23

Abbreviations: AE, antioxidant enzyme; BER, base excision repair; CI, confidence interval; GRS, genetic risk score; MAP, markers of adenomatous polyps; NSAID, non-steroidal anti-inflammatory drug; OBS, oxidative balance score; Prop. Diff., proportional difference; Ref., reference.

^a^ Geometric means, 95% confidence intervals, and *p*-values from general linear models; adjusted for total energy intake, sex, current hormone replacement therapy use, education (less than high school, high school degree, college graduate or higher), regular NSAID, use (<1/wk, ≥1/wk).

^b^ OBS, comprised 17 anti- and pro-oxidant dietary and lifestyle exposures (see text); a higher score represents a higher balance of anti-relative to pro-oxidant exposures.

^c^ Unequal sample sizes in tertiles due to ranking ties; differences in the numbers of participants due to availability of blood samples for assays.

^d^ Proportional difference calculated as (comparison group mean - reference group mean)/(reference group mean) x 100%.

^e^ BER GRS, based on 12 SNPs, in 5 BER, genes; see complete list of genes and SNPs, in the text and Supplementary Table S4; a higher GRS indicates a higher number of higher relative to lower risk alleles.

^f^ *P*_*interaction*_ from the interaction term for dichotomized GRS*OBS, tertiles in general linear models.

^g^ AE GRS, based on 4 SNPs, in the *GSTP1* gene; see complete list of SNPs, in the text and Supplementary Table S3; a higher GRS indicates a higher number of higher relative to lower risk alleles.

**FIGURE 1:**
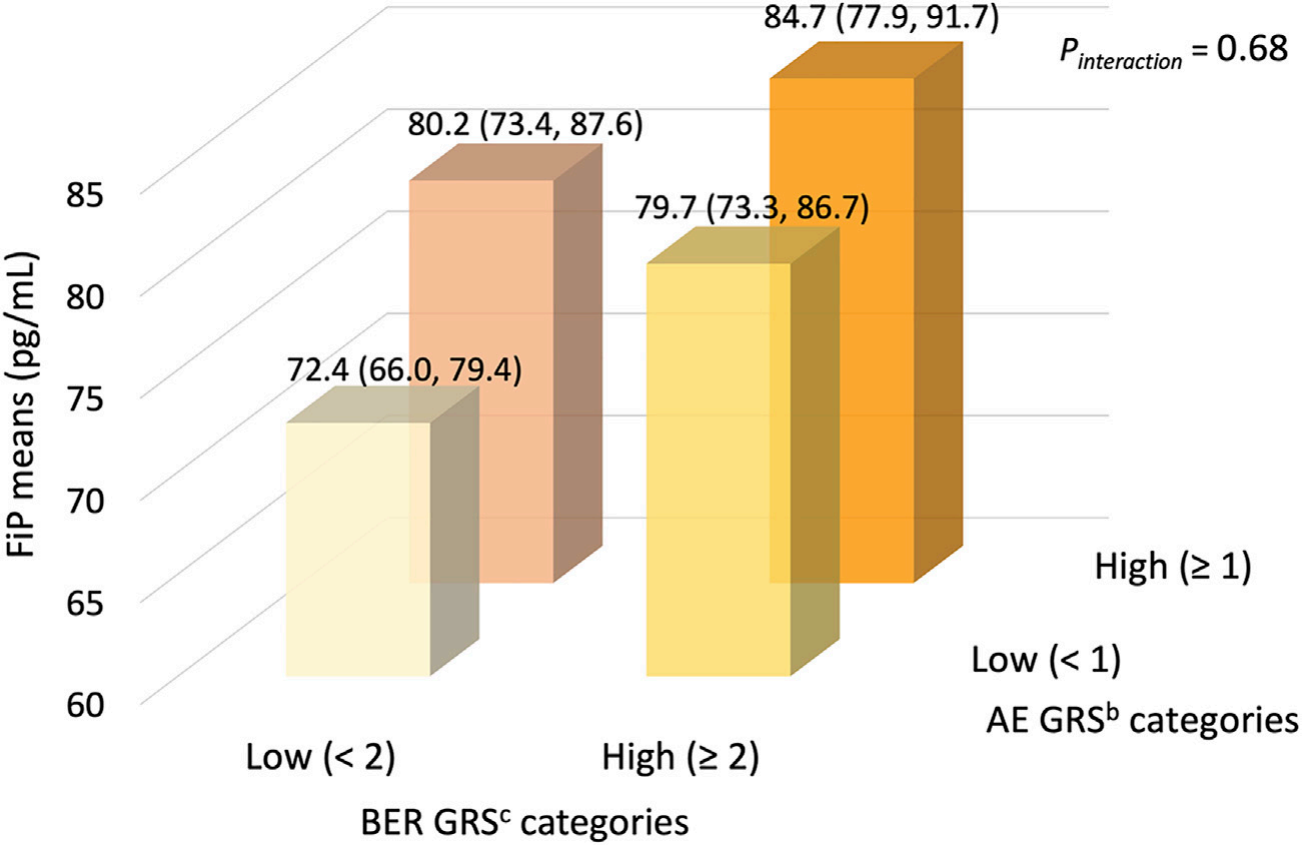
Mean^a^ plasma F_2_-isoprostanes concentrations (95% CIs), according to joint dichotomized categories of antioxidant enzyme and DNA base excision repair GRS, in the pooled MAP I and MAP II cross-sectional studies (*n* = 245). The joint lowest oxidative stress-specific AE/BER GRS category (*n* = 53) considered the reference group (the hypothesized lowest risk group). Compared to the reference group, the mean FiP concentrations in the joint low/high (*n* = 58), the joint high/low (*n* = 53), and the joint high/high AE/BER GRS category (*n* = 81) were, proportionately, 10.2% (*p* = 0.13), 10.8% (*p* = 0.12), and 16.4% (*p* = 0.01) higher, respectively. Abbreviations: AE, antioxidant enzyme; BER, base excision repair; CI, confidence interval; FiP, plasma F_2_-isoprostanes concentration; GRS, genetic risk score; MAP, Markers of Adenomatous Polyps. ^a^ Geometric means (95% CIs), adjusted for sex and the oxidative balance score (OBS). ^b^ AE GRS based on 4 SNPs in the *GSTP1* AE gene; see complete list of SNPs in the text and Supplementary Table S3. ^c^ BER GRS based on 12 SNPs in 5 BER genes; see complete list of genes and SNPs in the text and Supplementary Table S5.

We summarize the multivariable-adjusted mean plasma FiP concentrations in the joint dichotomized oxidative stress-specific AE/BER GRS categories in Figure 1. We considered the joint lowest oxidative stress-specific AE/BER GRS category to be the reference group (the hypothesized lowest risk group). Although we found no statistically significant interaction between the oxidative stress-specific AE and BER GRS, we noted that the highest mean FiP concentration was estimated to be among those in the joint highest oxidative stress-specific AE/BER GRS category; the mean FiP concentration among those in this category was, proportionately, 16.4% higher than among those in the joint lowest oxidative stress-specific AE/BER GRS category (*p* = 0.01).

## 4 Discussion

The findings from the present pilot study suggest that collective genotypes of certain DNA BER genes (*FEN1*, *MUTYH*, *OGG1*, *TDG*, and *XRCC1*), and possibly of the *GSTP1* AE gene, may be associated with systemic oxidative stress, as reflected by plasma FiP concentrations. Although we did not detect statistically significant interactions in this small study, we observed patterns that suggested that the oxidative stress-specific AE and BER GRS may 1) modestly modify the association of the OBS (representing the balance of the collective anti-relative to pro-oxidant dietary and lifestyle exposures) with systemic oxidative stress, and 2) modestly interact with each other, such that those with a higher relative to a lower joint oxidative stress-specific BER/AE GRS may have modestly higher systematic oxidative stress.

Previous basic science studies provide biological plausibility for investigating possible associations of AE and DNA BER genotypes with oxidative stress. Enzymatic antioxidant defense and base excision repair mechanisms contribute to regulating ROS levels in humans (Sun, 1990; Seeberg et al., 1995; Mates, 2000), and excess ROS production leads to oxidative stress (Storz and Imlayt, 1999; Sies, 2000). Antioxidant enzymes neutralize ROS-induced excessive cellular oxidation effects (Yang and Lee, 2015). For example, *GSTP1*, the gene included in our oxidative stress-specific AE GRS, plays an important role in detoxification of electrophilic metabolites and protection against ROS-induced damage (Henderson et al., 1998; Moyer et al., 2008). DNA BER, one of the major DNA damage repair pathways, is the main oxidative DNA damage repair pathway (Seeberg et al., 1995; Barzilai and Yamamoto, 2004). Previous studies suggested that DNA damage may contribute to ROS generation (Salmon et al., 2004; Rowe et al., 2008; Kang et al., 2012). For example, two *in vitro* studies found that in the absence of exposure to exogenous agents, intracellular ROS levels increased in BER-deficient cells relative to those in BER-proficient cells (Salmon et al., 2004; Rowe et al., 2008). Examples from the genes in our oxidative stress-specific BER GRS include: *XRCC1* contributes to single-stranded DNA break repair (Duell et al., 2000; Thompson and West, 2000); *TDG* repairs G/T and G/U mismatches *via* removing thymine and uracil moieties (He et al., 2011; Wu and Zhang, 2017); *OGG1* (Ba et al., 2014) and *MUTYH* (Sampson et al., 2005; Nielsen et al., 2011) contribute to repairing ROS-induced DNA base lesions *via* removing the mismatched 8-dihydro-8-oxoguanine adenine; and *FEN1* is involved in lagging-strand DNA synthesis and double-stranded DNA repair (Harrington and Lieber, 1994; Kucherlapati et al., 2002).

Recently, GRS (also known as polygenic risk scores [PRS]), have been used as tools for investigating risk for multiple diseases (Wang et al., 2017; Chalmer et al., 2018; Korologou-Linden et al., 2019a; Korologou-Linden et al., 2019b; Leonenko et al., 2019; Mavaddat et al., 2019; Watt et al., 2019; Zheutlin et al., 2019; Li et al., 2020; Mosley et al., 2020; Sipeky et al., 2020), thus supporting the idea of investigating associations of GRS with various outcomes. To our knowledge, there is only one reported investigation of an association of a GRS with oxidative stress (Kim et al., 2018). In a cross-sectional analysis of data from that study among Korean adults (*n* = 2,113), the authors constructed a GRS based on 25 SNPs in genes associated with higher susceptibility to having impaired fasting glucose control and Type 2 diabetes mellitus, and found statistically significant positive correlations between the GRS and two oxidative stress biomarkers (urinary FiP, *r* = 0.10, and plasma malondialdehyde, *r* = 0.13; both *p* < 0.001) (Kim et al., 2018). We found no reported investigations of an AE GRS with a disease outcome and only one (Wang et al., 2017) of a BER GRS. That study pooled data from three colonoscopy-based case-control studies (*n* = 408 adenoma cases, 604 controls), and calculated a BER GRS comprising 65 individual SNPs in 15 BER genes that were different from ours (Wang et al., 2017); among participants in the highest relative to the lowest BER GRS tertile, colorectal adenoma risk was substantially, statistically significantly higher (Wang et al., 2017).

BER genetic variants as potential effect modifiers of associations of anti- and pro-oxidant diet and lifestyle exposures with colorectal adenoma risk were investigated in several studies (Corral et al., 2013; Wang et al., 2017), thus supporting the possibility that BER GRS may modify associations of environmental exposures with various outcomes. However, our study is the first to report such potential effect modification in relation to an oxidative stress biomarker. In the three pooled case-control studies mentioned above, the authors also calculated an equal-weight OBS (a higher score reflected a higher balance of antioxidant relative to pro-oxidant dietary and lifestyle exposures); their findings suggested that having more BER risk variants combined with a lower OBS was associated with higher risk of colorectal adenoma than were either having more variants plus a higher OBS or having fewer genetic variants plus a lower OBS (Wang et al., 2017). A matched case-control study’s findings (*n* = 677 adenoma cases, 691 controls) also suggested that BER genes’ SNPs (the investigated SNPs differed those in our study) may modify associations of dietary folate, alcohol, and smoking with colorectal adenoma (Corral et al., 2013).

Ours is the first report of a joint/combined analysis to assess potential AE GRS-BER GRS interaction in relation to systemic oxidative stress. Although the *P*_*interaction*_ was not statistically significant in this small, preliminary study, the pattern of findings from the joint/combined analysis was consistent with a synergistic interaction. Future studies to assess potential AE GRS-BER GRS interaction in relation to oxidative stress in larger study populations are warranted.

Our study’s strengths include the extensive collection and assessment of dietary, lifestyle, and medical data as potential confounding factors, high quality laboratory measurements, and inclusion of both women and men. Also, to our knowledge, this is the first report of 1) associations of AE and BER GRS, separately and jointly, with an oxidative stress biomarker in humans, and 2) AE and BER GRS as potential effect modifiers of associations of collective dietary and lifestyle exposures with an oxidative stress biomarker.

Our study also has several limitations. First, we calculated the GRS based on tagSNPs in only AE and BER genes in a relatively small study population, rather than in a genome-wide association study in a large population, and multiple comparisons were involved. However, to our knowledge, ours is the first study to report associations of GRS with an oxidative stress biomarker, providing needed preliminary data and support for future investigations. Second, although our tagSNP approach comprehensively covered all common SNP variation in AE and BER genes, we could not investigate rare variations (MAF ≤5%); thus, we may have excluded potential influential SNPs. A third limitation includes the general limitations of using FFQs for assessing diet, such as recall error and limited food choices. However, we used a well-known, validated FFQ (Willett et al., 1985), and in our study, since participants reported dietary and lifestyle exposures before FiP was measured, recall error would likely be non-differential and thus be expected to attenuate the OBS results. Fourth, FiP, a measure of lipid peroxidation, was our only biomarker of oxidative stress; we had no measures of protein oxidation, oxidative DNA damage, or redox potential. However, FiP is one of the most reliably-measured, widely-used biomarkers of systemic oxidative stress in epidemiologic studies. Other limitations include the relatively small sample size—especially for stratified analyses—and that we only included White participants who went for outpatient colonoscopies, thus potentially limiting the generalizability of our findings.

In conclusion, our findings, in context with previous literature, suggest that combinations of genotypes of DNA BER genes, and possibly of AE genes, may be associated with systemic oxidative stress in humans. Our pilot study supports further investigations, using multiple biomarkers of oxidative stress, of oxidative stress-specific individual BER and AE GRS and potential AE GRS-BER GRS and AE and BER GRS-OBS interactions in larger, general populations.

## Data Availability

Original datasets are available in a publicly accessible repository: https://github.com/ZLING823/Dataset-for-GRS-OBS-project.git.
