# Supplementary material for: Antioxidant enzyme and DNA base repair genetic risk scores’ associations with systemic oxidative stress biomarker in pooled cross-sectional studies
Source: Front Aging. 2023 Apr 21;4:1000166. doi: 10.3389/fragi.2023.1000166 (PMC10161255; doi:10.3389/fragi.2023.1000166)
Supplement: Supplementary file 1 [file Table1.DOCX]

Supplementary Materials

Article Title: Associations of Antioxidant Enzyme and DNA Base Repair Genetic Risk Scores with Biomarker of Systemic Oxidative Stress in Pooled Cross-Sectional Studies

Authors: Ziling Mao^1^, Abigail L. H. Gray^1^, Myron D. Gross^2^, Bharat Thyagarajan^2^, Roberd M. Bostick^1,3^*

^1^Department of Epidemiology, Rollins School of Public Health, Emory University, Atlanta, Georgia, USA.

^2^Department of Laboratory Medicine and Pathology, University of Minnesota, USA.

^3^Winship Cancer Institute, Emory University, Atlanta, Georgia, USA.

*** Correspondence:**Roberd M. Bostick, MD, MPH
rmbosti@emory.edu

**Supplementary Table S1.** Antioxidant enzyme genes and SNPs investigated.

| **Gene** | **SNP rs ID** | **RefSNP alleles (strand direction)** | **Minor allele**^a^ |
| --- | --- | --- | --- |
| *CAT* | rs1001179 | A/G (REV) | T |
| *CAT* | rs7947841 | A/G (FWD) | A |
| *CAT* | rs499406 | A/C/G (REV) | T |
| *CAT* | rs566979 | A/G/T (REV) | C |
| *CAT* | rs16925614 | C/T (FWD) | T |
| *CAT* | rs11032703 | C/T (FWD) | T |
| *CAT* | rs11604331 | A/G (FWD) | G |
| *CAT* | rs525938 | A/G (REV) | C |
| *CAT* | rs7104301 | A/G (FWD) | G |
| *CAT* | rs12272630 | C/G (FWD) | C |
| *CAT* | rs7943316 | A/T (FWD) | T |
|  |  |  |  |
| *GSTP1* | rs4147581 | C/G (FWD) | G |
| *GSTP1* | rs1138272 | C/T (FWD) | T |
| *GSTP1* | rs749174 | C/T (REV) | A |
| *GSTP1* | rs1695 | A/G (FWD) | G |
| *GSTP1* | rs762803 | A/C (FWD) | A |
|  |  |  |  |
| *MnSOD* | rs5746151 | A/G (REV) | T |
| *MnSOD* | rs5746136 | A/G (REV) | T |
| *MnSOD* | rs4880 | C/T (REV) | G |
| *MnSOD* | rs6917589 | C/T (FWD) | C |
| *MnSOD* | rs8031 | A/T (REV) | T |
| *MnSOD* | rs2842980 | A/T (REV) | A |

Abbreviations: FWD, forward; ID, identifier; REV, reverse; SNP, single nucleotide polymorphism.

^a^ Data from 1000 Genomes Project.

**Supplementary Table S2.** DNA base excision repair pathway genes and SNPs investigated.

| **Gene** | **SNP rs ID** | **RefSNP alleles (strand direction)** | **Minor allele**^a^ |
| --- | --- | --- | --- |
| *APEX1* | rs3136814 | A/C (FWD) | C |
| *APEX1* | rs1130409 | A/G/T (FWD) | G |
| *APEX1* | rs1760944 | A/C (REV) | T |
|  |  |  |  |
| *FEN1* | rs412334 | A/G (REV) | T |
|  |  |  |  |
| *LIG1* | rs419664 | A/G/T (REV) | A |
| *LIG1* | rs156641 | A/G (REV) | T |
| *LIG1* | rs2288881 | A/G (REV) | T |
| *LIG1* | rs3730947 | A/G (REV) | T |
| *LIG1* | rs3731037 | C/T (REV) | A |
| *LIG1* | rs411073 | C/T (REV) | A |
| *LIG1* | rs3730908 | C/T (REV) | A |
| *LIG1* | rs20579 | C/G/T (REV) | A |
| *LIG1* | rs3730881 | C/T (REV) | A |
| *LIG1* | rs3730914 | C/T (REV) | A |
| *LIG1* | rs3731003 | C/T (REV) | A |
| *LIG1* | rs3730837 | A/G (REV) | C |
| *LIG1* | rs274862 | C/T (FWD) | C |
| *LIG1* | rs3730912 | A/C (REV) | T |
| *LIG1* | rs20580 | A/C (REV) | G |
|  |  |  |  |
| *LIG3* | rs3135974 | A/G (FWD) | A |
| *LIG3* | rs3135998 | A/G (FWD) | A |
| *LIG3* | rs3135989 | G/T (FWD) | G |
| *LIG3* | rs3135967 | A/G (FWD) | G |
| *LIG3* | rs2074516 | C/G (REV) | G |
|  |  |  |  |
| *MBD4* | rs3138360 | A/G (REV) | T |
| *MBD4* | rs10342 | A/G/T (REV) | T |
| *MBD4* | rs2005618 | C/T (REV) | G |
| *MBD4* | rs2311394 | C/T (REV) | G |
| *MBD4* | rs2307293 | C/G (REV) | G |
| *MBD4* | rs3138326 | A/T (REV) | A |
|  |  |  |  |
| *MPG* | rs3176415 | A/G (FWD) | G |
| *MPG* | rs2541622 | C/G/T (REV) | A |
| *MPG* | rs3176424 | A/G (FWD) | G |
|  |  |  |  |
| *MUTYH* | rs3219476 | G/T (REV) | A |
| *MUTYH* | rs3219484 | A/G (REV) | T |
| *MUTYH* | rs3219494 | A/G (REV) | T |
| *MUTYH* | rs3219463 | A/G (REV) | T |
| *MUTYH* | rs3219489 | C/G (REV) | T |
| *MUTYH* | rs3219493 | C/G (REV) | G |
|  |  |  |  |
| *OGG1* | rs125701 | A/G (FWD) | A |
| *OGG1* | rs1805373 | A/C/G (FWD) | A |
| *OGG1* | rs2072668 | C/G (FWD) | G |
| *OGG1* | rs3219008 | A/G (FWD) | G |
| *OGG1* | rs159153 | C/T (FWD) | C |
| *OGG1* | rs293795 | C/T (REV) | G |
|  |  |  |  |
| *PNKP* | rs3739206 | G/T (REV) | C |
| *PNKP* | rs2257103 | C/T (FWD) | T |
| *PNKP* | rs3739186 | A/T (REV) | T |
|  |  |  |  |
| *POLB* | rs2979896 | G/T (REV) | C |
| *POLB* | rs3136811 | C/G (FWD) | G |
| *POLB* | rs3136797 | C/G (FWD) | G |
|  |  |  |  |
| *SMUG1* | rs2233920 | G/T (REV) | A |
| *SMUG1* | rs3136386 | C/G (REV) | C |
| *SMUG1* | rs971 | C/T (FWD) | T |
| *SMUG1* | rs2279402 | C/T (REV) | G |
|  |  |  |  |
| *TDG* | rs3829301 | A/C (FWD) | C |
| *TDG* | rs4135113 | A/G/T (FWD) | A |
| *TDG* | rs2629768 | A/G (REV) | T |
| *TDG* | rs4135064 | C/T (FWD) | T |
| *TDG* | rs322107 | C/T (REV) | A |
| *TDG* | rs4135061 | A/G (FWD) | G |
| *TDG* | rs4135081 | A/G (FWD) | G |
| *TDG* | rs322109 | A/C/G (REV) | C |
| *TDG* | rs4135093 | C/T (FWD) | C |
| *TDG* | rs4135094 | C/T (FWD) | C |
| *TDG* | rs167715 | C/T (REV) | G |
|  |  |  |  |
| *UNG* | rs3219245 | G/T (FWD) | T |
| *UNG* | rs246079 | A/G (FWD) | A |
|  |  |  |  |
| *XRCC1* | rs939461 | A/C (FWD) | C |
| *XRCC1* | rs3213247 | G/T (REV) | A |
| *XRCC1* | rs939460 | A/G (FWD) | A |
| *XRCC1* | rs25487 | A/G (REV) | T |
| *XRCC1* | rs25489 | A/C/G (REV) | T |
| *XRCC1* | rs1001581 | C/T (FWD) | T |
| *XRCC1* | rs2307191 | C/T (REV) | A |
| *XRCC1* | rs3213403 | A/G (REV) | C |
| *XRCC1* | rs915927 | A/C/G/T (REV) | C |
| *XRCC1* | rs3213255 | C/T (REV) | G |
| *XRCC1* | rs25496 | C/T (REV) | G |

Abbreviations: FWD, forward; ID, identifier; REV, reverse; SNP, single nucleotide polymorphism.

^a^ Data from 1000 Genomes Project.

**Supplementary Table S3.**  Antioxidant enzyme genetic risk score genes and SNPs^a^ and their functions.

| **Gene** | **Functions** | **SNP rs ID** | **Consequence** |
| --- | --- | --- | --- |
| *GSTP1* | Detoxifies electrophilic metabolites and protects against ROS-induced damage (Henderson et al., 1998; Moyer et al., 2008) | rs4147581 | Intron Variant |
|  |  | rs749174 | Intron Variant |
|  |  | rs1695 | Missense Variant |
|  |  | rs762803 | Intron Variant |

Abbreviations: AE, antioxidant enzyme; GRS, genetic risk score; ID, identifier; SNP, single nucleotide polymorphism.

^a^ Genes and SNPs from **Supplementary Table S3** included in the AE GRS if in **Supplementary Table S5** the proportional mean differences in plasma F_2_-isoprostanes concentrations were > 5% plus *p* ≤ 0.05 or > 10% plus *p* ≤ 0.15.

**Supplementary Table S4.**  DNA base excision repair genetic risk score genes and SNPs^a^ and their functions.

| **Gene** | **Functions** | **SNP rs ID** | **Consequence** |
| --- | --- | --- | --- |
| *FEN1* | Involved in lagging-strand DNA synthesis and double-stranded DNA repair (Harrington and Lieber, 1994; Kucherlapati et al., 2002) | rs412334 | Upstream Variant |
|  |  |  |  |
| *MUTYH* | Contributes to repairing oxidative damage by removing the mismatched 8-oxoG adenine (Sampson et al., 2005; Nielsen et al., 2011) | rs3219476 | Intron Variant |
|  |  | rs3219489 | Missense Variant |
|  |  |  |  |
| *OGG1* | Contributes to repairing ROS-induced DNA base lesions via removing the mismatched 8-oxoG (Ba et al., 2014) | rs125701 | 2KB Upstream Variant |
|  |  | rs2072668 | Intron Variant |
|  |  | rs3219008 | Intron Variant |
|  |  | rs159153 | 2KB Upstream Variant |
|  |  | rs293795 | Intron Variant |
|  |  |  |  |
| *TDG* | Involved in DNA demethylation; repairs G/T and G/U mismatches via removing thymine and uracil moieties (He et al., 2011; Wu and Zhang, 2017) | rs4135061 | Intron Variant |
|  |  | rs4135094 | Intron Variant |
|  |  |  |  |
| *XRCC1* | Repairs single-stranded DNA breaks (Duell et al., 2000; Thompson and West, 2000) | rs939460 | Intron Variant |
|  |  | rs1001581 | Intron Variant |

Abbreviations: ID, identifier; SNP, single nucleotide polymorphism; 8-oxoG, 8-dihydro-2′ -deoxyguanosine.

^a^ Genes and SNPs from **Supplementary Table S3** included in the AE GRS if in **Supplementary Table S6** the proportional mean differences in plasma F_2_-isoprostanes concentrations were > 5% plus *p* ≤ 0.05 or > 10% plus *p* ≤ 0.15.

**Supplementary Table S5.**  Distributions of antioxidant enzyme genotypes, and mean plasma F_2_-isoprostanes (pg/mL) concentrations according to the genotypes, in the pooled MAP I and MAP II cross-sectional studies (*n* = 245)^a^.

| **Gene** | **SNP** | **Genotype** | **Weight** | ***n*** | **HWE** | | |  | **Geometric means** | | | **Prop. diff.**^c^ **(%)** | ***p*** |
| --- | --- | --- | --- | --- | --- | --- | --- | --- | --- | --- | --- | --- | --- |
|  |  |  |  |  | **Expected *n***^b^ | **χ^2^** | ***p*** |  | **Mean** | **95% CL** | |  |  |
|  |  |  |  |  |  |  |  |  |  | **LL** | **UL** |  |  |
| ***CAT*** | **rs1001179** |  |  |  |  |  |  |  |  |  |  |  |  |
|  |  | **Missing** | 0 | 3 |  |  |  |  |  |  |  |  |  |
|  |  | **GG** | 0 | 141 | 143.7 |  |  |  | 78.8 | 74.2 | 83.6 |  |  |
|  |  | **GA** | 1 | 91 | 85.5 |  |  |  | 79.9 | 74.3 | 86.0 |  |  |
|  |  | **AA** | 2 | 10 | 12.7 | 0.98 | 0.32 |  | 81.2 | 65.0 | 101.4 | 3.0 | 0.93 |
|  |  |  |  |  |  |  |  |  |  |  |  |  |  |
| ***CAT*** | **rs7947841**^d^ |  |  |  |  |  |  |  |  |  |  |  |  |
|  |  | **Missing** | 0 | 3 |  |  |  |  |  |  |  |  |  |
|  |  | **GG** | 0 | 210 | 208.3 |  |  |  | 79.8 | 76.1 | 83.8 |  |  |
|  |  | **GA** | 1 | 29 | 32.5 |  |  |  | 74.4 | 65.3 | 84.7 |  |  |
|  |  | **AA** | 2 | 3 | 1.3 | 2.76 | 0.10 |  | 93.0 | 61.9 | 139.7 | 16.5 | 0.44 |
|  |  | **GA + AA** |  | 32 |  |  |  |  | 75.9 | 67.0 | 85.9 | -5.0 | 0.45 |
|  |  |  |  |  |  |  |  |  |  |  |  |  |  |
| ***CAT*** | **rs499406** |  |  |  |  |  |  |  |  |  |  |  |  |
|  |  | **Missing** | 1 | 3 |  |  |  |  |  |  |  |  |  |
|  |  | **GG** | 0 | 90 | 71.5 |  |  |  | 76.5 | 71.1 | 82.4 |  |  |
|  |  | **GA** | 1 | 106 | 143.0 |  |  |  | 82.8 | 77.4 | 88.6 |  |  |
|  |  | **AA** | 2 | 90 | 71.5 | 19.15 | < 0.01 |  | 77.0 | 69.5 | 85.3 | 0.6 | 0.24 |
|  |  |  |  |  |  |  |  |  |  |  |  |  |  |
| ***CAT*** | **rs566979** |  |  |  |  |  |  |  |  |  |  |  |  |
|  |  | **Missing** | 1 | 3 |  |  |  |  |  |  |  |  |  |
|  |  | **TT** | 0 | 89 | 91.7 |  |  |  | 77.9 | 72.3 | 83.9 |  |  |
|  |  | **TG** | 1 | 120 | 114.5 |  |  |  | 81.4 | 76.4 | 86.8 |  |  |
|  |  | **GG** | 2 | 33 | 35.7 | 0.55 | 0.46 |  | 75.8 | 67.1 | 85.5 | -2.7 | 0.49 |
|  |  |  |  |  |  |  |  |  |  |  |  |  |  |
| ***CAT*** | **rs16925614**^d^ |  |  |  |  |  |  |  |  |  |  |  |  |
|  |  | **Missing** | 0 | 3 |  |  |  |  |  |  |  |  |  |
|  |  | **CC** | 0 | 181 | 181.4 |  |  |  | 79.7 | 75.7 | 84.0 |  |  |
|  |  | **CT** | 1 | 57 | 56.3 |  |  |  | 78.1 | 71.2 | 85.7 |  |  |
|  |  | **TT** | 2 | 4 | 4.4 | 0.04 | 0.84 |  | 77.5 | 54.7 | 109.9 | -2.8 | 0.92 |
|  |  | **CT + TT** |  | 61 |  |  |  |  | 78.1 | 71.4 | 85.4 | -2.1 | 0.69 |
|  |  |  |  |  |  |  |  |  |  |  |  |  |  |
| ***CAT*** | **rs11032703**^d^ |  |  |  |  |  |  |  |  |  |  |  |  |
|  |  | **Missing** | 0 | 4 |  |  |  |  |  |  |  |  |  |
|  |  | **CC** | 0 | 190 | 191.9 |  |  |  | 79.5 | 75.6 | 83.7 |  |  |
|  |  | **CT** | 1 | 51 | 47.2 |  |  |  | 78.5 | 71.1 | 86.7 |  |  |
|  |  | **TT** | 2 | 1 | 2.9 | 1.57 | 0.21 |  | 78.6 | 39.0 | 158.5 | -1.2 | 0.97 |
|  |  | **CT + TT** |  | 52 |  |  |  |  | 78.5 | 71.1 | 86.6 | -1.3 | 0.82 |
|  |  |  |  |  |  |  |  |  |  |  |  |  |  |
| ***CAT*** | **rs11604331** |  |  |  |  |  |  |  |  |  |  |  |  |
|  |  | **Missing** | 1 | 3 |  |  |  |  |  |  |  |  |  |
|  |  | **AA** | 0 | 104 | 101.2 |  |  |  | 77.3 | 72.2 | 82.8 |  |  |
|  |  | **AG** | 1 | 105 | 110.6 |  |  |  | 82.6 | 77.2 | 88.5 |  |  |
|  |  | **GG** | 2 | 33 | 30.2 | 0.62 | 0.43 |  | 75.3 | 66.8 | 85.0 | -2.6 | 0.27 |
|  |  |  |  |  |  |  |  |  |  |  |  |  |  |
| ***CAT*** | **rs525938** |  |  |  |  |  |  |  |  |  |  |  |  |
|  |  | **Missing** | 0 | 3 |  |  |  |  |  |  |  |  |  |
|  |  | **AA** | 0 | 129 | 127.3 |  |  |  | 80.1 | 75.4 | 85.2 |  |  |
|  |  | **AG** | 1 | 93 | 96.5 |  |  |  | 79.6 | 74.0 | 85.6 |  |  |
|  |  | **GG** | 2 | 20 | 18.3 | 0.31 | 0.58 |  | 72.7 | 62.1 | 85.1 | -9.3 | 0.52 |
|  |  |  |  |  |  |  |  |  |  |  |  |  |  |
| ***CAT*** | **rs7104301** |  |  |  |  |  |  |  |  |  |  |  |  |
|  |  | **Missing** | 0 | 4 |  |  |  |  |  |  |  |  |  |
|  |  | **AA** | 0 | 117 | 120.6 |  |  |  | 77.6 | 72.7 | 82.7 |  |  |
|  |  | **AG** | 1 | 107 | 99.8 |  |  |  | 82.0 | 76.6 | 87.8 |  |  |
|  |  | **GG** | 2 | 17 | 20.6 | 1.27 | 0.26 |  | 73.5 | 62.1 | 87.1 | -5.2 | 0.35 |
|  |  |  |  |  |  |  |  |  |  |  |  |  |  |
| ***CAT*** | **rs12272630**^d^ |  |  |  |  |  |  |  |  |  |  |  |  |
|  |  | **Missing** | 0 | 3 |  |  |  |  |  |  |  |  |  |
|  |  | **GG** | 0 | 232 | 231.1 |  |  |  | 79.1 | 75.5 | 82.8 |  |  |
|  |  | **GC** | 1 | 9 | 10.8 |  |  |  | 85.2 | 67.4 | 107.7 |  |  |
|  |  | **CC** | 2 | 1 | 0.1 | 6.41 | 0.01 |  | 84.1 | 41.4 | 171.2 | 6.4 | 0.81 |
|  |  | **GC + CC** |  | 10 |  |  |  |  | 85.1 | 68.2 | 106.2 | 7.6 | 0.12 |
|  |  |  |  |  |  |  |  |  |  |  |  |  |  |
| ***CAT*** | **rs7943316** |  |  |  |  |  |  |  |  |  |  |  |  |
|  |  | **Missing** | 1 | 3 |  |  |  |  |  |  |  |  |  |
|  |  | **TT** | 0 | 113 | 113.2 |  |  |  | 78.7 | 73.9 | 83.9 |  |  |
|  |  | **TA** | 1 | 105 | 104.6 |  |  |  | 78.8 | 73.9 | 84.1 |  |  |
|  |  | **AA** | 2 | 24 | 24.2 | 0.00 | 0.96 |  | 73.6 | 64.1 | 84.5 | -6.5 | 0.46 |
|  |  |  |  |  |  |  |  |  |  |  |  |  |  |
| ***GSTP1*** | **rs4147581** |  |  |  |  |  |  |  |  |  |  |  |  |
|  |  | **Missing** | 1 | 11 |  |  |  |  |  |  |  |  |  |
|  |  | **CC** | 0 | 70 | 70.0 |  |  |  | 81.1 | 74.6 | 88.2 |  |  |
|  |  | **CG** | 1 | 116 | 116.0 |  |  |  | 81.1 | 75.9 | 86.5 |  |  |
|  |  | **GG** | 2 | 48 | 48.0 | 0.00 | 1.00 |  | 71.9 | 65.0 | 79.5 | -11.4 | 0.11 |
|  |  |  |  |  |  |  |  |  |  |  |  |  |  |
| ***GSTP1*** | **rs1138272**^d^ |  |  |  |  |  |  |  |  |  |  |  |  |
|  |  | **Missing** | 0 | 2 |  |  |  |  |  |  |  |  |  |
|  |  | **CC** | 0 | 203 | 201.9 |  |  |  | 78.8 | 75.1 | 82.7 |  |  |
|  |  | **CT** | 1 | 37 | 39.2 |  |  |  | 81.3 | 72.6 | 91.0 |  |  |
|  |  | **TT** | 2 | 3 | 1.9 | 0.76 | 0.38 |  | 124.2 | 83.4 | 184.8 | 57.6 | 0.08 |
|  |  | **CT + TT** |  | 40 |  |  |  |  | 83.9 | 75.2 | 93.6 | 6.5 | 0.47 |
|  |  |  |  |  |  |  |  |  |  |  |  |  |  |
| ***GSTP1*** | **rs749174** |  |  |  |  |  |  |  |  |  |  |  |  |
|  |  | **Missing** | 1 | 0 |  |  |  |  |  |  |  |  |  |
|  |  | **CC** | 0 | 108 | 107.8 |  |  |  | 74.7 | 69.9 | 79.8 |  |  |
|  |  | **CT** | 1 | 109 | 109.4 |  |  |  | 83.8 | 78.4 | 89.5 |  |  |
|  |  | **TT** | 2 | 28 | 27.8 | 0.00 | 0.95 |  | 81.3 | 71.4 | 92.6 | 8.9 | 0.05 |
|  |  |  |  |  |  |  |  |  |  |  |  |  |  |
| ***GSTP1*** | **rs1695** |  |  |  |  |  |  |  |  |  |  |  |  |
|  |  | **Missing** | 0 | 0 |  |  |  |  |  |  |  |  |  |
|  |  | **AA** | 0 | 111 | 108.4 |  |  |  | 74.7 | 70.0 | 79.8 |  |  |
|  |  | **AG** | 1 | 104 | 109.1 |  |  |  | 84.2 | 78.7 | 90.1 |  |  |
|  |  | **GG** | 2 | 30 | 27.4 | 0.54 | 0.46 |  | 80.6 | 71.1 | 91.4 | 7.9 | 0.04 |
|  |  |  |  |  |  |  |  |  |  |  |  |  |  |
| ***GSTP1*** | **rs762803** |  |  |  |  |  |  |  |  |  |  |  |  |
|  |  | **Missing** | 1 | 3 |  |  |  |  |  |  |  |  |  |
|  |  | **CC** | 0 | 79 | 75.9 |  |  |  | 73.5 | 68.0 | 79.5 |  |  |
|  |  | **CA** | 1 | 113 | 119.3 |  |  |  | 81.8 | 76.7 | 87.3 |  |  |
|  |  | **AA** | 2 | 50 | 46.9 | 0.67 | 0.41 |  | 83.2 | 75.4 | 91.8 | 13.2 | 0.07 |
|  |  |  |  |  |  |  |  |  |  |  |  |  |  |
| ***MnSOD*** | **rs5746151**^d^ |  |  |  |  |  |  |  |  |  |  |  |  |
|  |  | **Missing** | 0 | 0 |  |  |  |  |  |  |  |  |  |
|  |  | **GG** | 0 | 216 | 215.9 |  |  |  | 79.3 | 75.6 | 83.1 |  |  |
|  |  | **GA** | 1 | 28 | 28.2 |  |  |  | 79.8 | 70.0 | 91.1 |  |  |
|  |  | **AA** | 2 | 1 | 0.9 | 0.01 | 0.93 |  | 81.0 | 40.3 | 162.7 | 2.1 | 0.99 |
|  |  | **GA + AA** |  | 29 |  |  |  |  | 79.9 | 70.2 | 90.9 | 0.7 | 0.92 |
|  |  |  |  |  |  |  |  |  |  |  |  |  |  |
| ***MnSOD*** | **rs5746136** |  |  |  |  |  |  |  |  |  |  |  |  |
|  |  | **Missing** | 0 | 3 |  |  |  |  |  |  |  |  |  |
|  |  | **GG** | 0 | 125 | 122.2 |  |  |  | 81.4 | 76.5 | 86.6 |  |  |
|  |  | **GA** | 1 | 94 | 99.5 |  |  |  | 77.1 | 71.8 | 82.8 |  |  |
|  |  | **AA** | 2 | 23 | 20.2 | 0.74 | 0.39 |  | 78.9 | 68.2 | 91.2 | -3.1 | 0.53 |
|  |  |  |  |  |  |  |  |  |  |  |  |  |  |
| ***MnSOD*** | **rs4880** |  |  |  |  |  |  |  |  |  |  |  |  |
|  |  | **Missing** | 1 | 9 |  |  |  |  |  |  |  |  |  |
|  |  | **CC** | 0 | 57 | 67.3 |  |  |  | 82.9 | 75.5 | 90.9 |  |  |
|  |  | **CT** | 1 | 138 | 117.5 |  |  |  | 77.9 | 73.3 | 82.6 |  |  |
|  |  | **TT** | 2 | 41 | 51.3 | 7.22 | 0.01 |  | 78.0 | 69.9 | 87.0 | -5.9 | 0.52 |
|  |  |  |  |  |  |  |  |  |  |  |  |  |  |
| ***MnSOD*** | **rs6917589** |  |  |  |  |  |  |  |  |  |  |  |  |
|  |  | **Missing** | 0 | 3 |  |  |  |  |  |  |  |  |  |
|  |  | **TT** | 0 | 142 | 142.2 |  |  |  | 81.5 | 76.9 | 86.4 |  |  |
|  |  | **TC** | 1 | 87 | 86.6 |  |  |  | 74.8 | 69.4 | 80.5 |  |  |
|  |  | **CC** | 2 | 13 | 13.2 | 0.00 | 0.95 |  | 87.0 | 71.8 | 105.5 | 6.7 | 0.12 |
|  |  |  |  |  |  |  |  |  |  |  |  |  |  |
| ***MnSOD*** | **rs8031** |  |  |  |  |  |  |  |  |  |  |  |  |
|  |  | **Missing** | 1 | 4 |  |  |  |  |  |  |  |  |  |
|  |  | **TT** | 0 | 60 | 61.8 |  |  |  | 74.5 | 68.1 | 81.5 |  |  |
|  |  | **TA** | 1 | 124 | 120.5 |  |  |  | 80.3 | 75.4 | 85.4 |  |  |
|  |  | **AA** | 2 | 57 | 58.8 | 0.21 | 0.65 |  | 82.5 | 75.2 | 90.4 | 10.7 | 0.26 |
|  |  |  |  |  |  |  |  |  |  |  |  |  |  |
| ***MnSOD*** | **rs2842980**^d^ |  |  |  |  |  |  |  |  |  |  |  |  |
|  |  | **Missing** | 0 | 3 |  |  |  |  |  |  |  |  |  |
|  |  | **AA** | 0 | 151 | 156.3 |  |  |  | 80.6 | 76.2 | 85.3 |  |  |
|  |  | **AT** | 1 | 87 | 76.4 |  |  |  | 77.5 | 71.9 | 83.5 |  |  |
|  |  | **TT** | 2 | 4 | 9.3 | 4.71 | 0.03 |  | 69.9 | 49.4 | 99.1 | -13.3 | 0.55 |
|  |  | **AT + TT** |  | 91 |  |  |  |  | 77.1 | 71.7 | 83.0 | -4.3 | 0.35 |

Abbreviations: CL, confidence limit; Diff., difference; HWE, Hardy-Weinberg Equilibrium; LL, lower limit; MAP, Markers of Adenomatous Polyps; Prop. diff., proportional difference; SNP, single nucleotide polymorphism; UL, upper limit.

^a^ Geometric means, 95% confidence limits, and *p*-values from general linear models, adjusted for sex (male/female) and body mass index (continuous).

^b^ Based on minor allele frequency in the European population in the 1000 Genomes Project Phase 3 (ensembl.org OR ncbi.nlm.nih.gov/projects/SNP).

^c^ Proportional difference, in percent, between mean plasma F_2_-isoprostanes concentration among those with a variant genotype relative to those with the common homozygous genotype; i.e.: (variant mean - common mean) / common mean x 100%.

^d^ Heterozygous and/or variant homozygous genotypes with ≤ 10 participants were combined.

**Supplementary Table S6.** Distributions of DNA base excision repair genotypes, and plasma F2-isoprostanes concentrations (pg/mL), according to the genotypes, in the pooled MAP I and MAP II cross-sectional studies (*n* = 245)^a^.

| **Gene** | **SNP** | **Genotype** | **Weight** | ***n*** | **HWE** | | |  | **Geometric means** | | | **Prop. diff.**^c^ **(%)** | ***p*** |
| --- | --- | --- | --- | --- | --- | --- | --- | --- | --- | --- | --- | --- | --- |
|  |  |  |  |  | **Expected n**^b^ | **χ^2^** | ***p*** |  | **Mean** | **95% CL** | |  |  |
|  |  |  |  |  |  |  |  |  |  | **LL** | **UL** |  |  |
| ***APEX1*** | **rs3136814** |  |  |  |  |  |  |  |  |  |  |  |  |
|  |  | **Missing** | 0 | 2 |  |  |  |  |  |  |  |  |  |
|  |  | **AA** | 0 | 130 | 130.3 |  |  |  | 79.6 | 76.1 | 83.4 | - |  |
|  |  | **AC** | 1 | 13 | 12.4 | 0.03 | 0.87 |  | 74.1 | 61.1 | 89.9 | -6.9 | 0.48 |
|  |  |  |  |  |  |  |  |  |  |  |  |  |  |
| ***APEX1*** | **rs1130409** |  |  |  |  |  |  |  |  |  |  |  |  |
|  |  | **Missing** | 1 | 3 |  |  |  |  |  |  |  |  |  |
|  |  | **GG** | 0 | 59 | 60.5 |  |  |  | 84.2 | 76.9 | 92.1 | - |  |
|  |  | **GT** | 1 | 124 | 121.0 |  |  |  | 77.4 | 72.8 | 82.4 | -8.0 |  |
|  |  | **TT** | 2 | 59 | 60.5 | 0.15 | 0.70 |  | 79.7 | 72.8 | 87.3 | -5.3 | 0.33 |
|  |  |  |  |  |  |  |  |  |  |  |  |  |  |
| ***APEX1*** | **rs1760944** |  |  |  |  |  |  |  |  |  |  |  |  |
|  |  | **Missing** | 1 | 16 |  |  |  |  |  |  |  |  |  |
|  |  | **CC** | 0 | 81 | 80.8 |  |  |  | 84.3 | 77.9 | 91.1 | - |  |
|  |  | **CA** | 1 | 110 | 110.5 |  |  |  | 76.3 | 71.4 | 81.6 | -9.4 |  |
|  |  | **AA** | 2 | 38 | 37.8 | 0.00 | 0.95 |  | 79.5 | 70.9 | 89.1 | -5.6 | 0.17 |
|  |  |  |  |  |  |  |  |  |  |  |  |  |  |
| ***FEN1*** | **rs412334**^d^ |  |  |  |  |  |  |  |  |  |  |  |  |
|  |  | **Missing** | 0 | 13 |  |  |  |  |  |  |  |  |  |
|  |  | **GG** | 0 | 163 | 166.4 |  |  |  | 77.3 | 73.2 | 81.6 | - |  |
|  |  | **GA** | 1 | 67 | 60.1 |  |  |  | 85.1 | 78.2 | 92.7 | 10.1 |  |
|  |  | **AA** | 2 | 2 | 5.4 | 3.02 | 0.08 |  | 98.9 | 60.4 | 162.0 | 28.0 | 0.12 |
|  |  | **GA+AA** |  | 69 |  |  |  |  | 85.5 | 78.7 | 93.0 | 10.6 | 0.05 |
|  |  |  |  |  |  |  |  |  |  |  |  |  |  |
| ***LIG1*** | **rs419664** |  |  |  |  |  |  |  |  |  |  |  |  |
|  |  | **Missing** | 1 | 2 |  |  |  |  |  |  |  |  |  |
|  |  | **GG** | 0 | 79 | 76.7 |  |  |  | 78.0 | 72.1 | 84.3 | - |  |
|  |  | **GT** | 1 | 115 | 119.6 |  |  |  | 81.2 | 76.1 | 86.6 | 4.2 |  |
|  |  | **TT** | 2 | 49 | 46.7 | 0.37 | 0.54 |  | 77.0 | 69.7 | 85.0 | -1.3 | 0.59 |
|  |  |  |  |  |  |  |  |  |  |  |  |  |  |
| ***LIG1*** | **rs156641** |  |  |  |  |  |  |  |  |  |  |  |  |
|  |  | **Missing** | 1 | 2 |  |  |  |  |  |  |  |  |  |
|  |  | **GG** | 0 | 96 | 92.0 |  |  |  | 80.6 | 75.0 | 86.5 | - |  |
|  |  | **GA** | 1 | 107 | 115.0 |  |  |  | 77.9 | 72.8 | 83.3 | -3.3 |  |
|  |  | **AA** | 2 | 40 | 36.0 | 1.19 | 0.28 |  | 82.4 | 73.8 | 92.0 | 2.2 | 0.64 |
|  |  |  |  |  |  |  |  |  |  |  |  |  |  |
| ***LIG1*** | **rs2288881**^d^ |  |  |  |  |  |  |  |  |  |  |  |  |
|  |  | **Missing** | 0 | 1 |  |  |  |  |  |  |  |  |  |
|  |  | **GG** | 0 | 222 | 221.5 |  |  |  | 79.3 | 75.7 | 83.1 | - |  |
|  |  | **GA** | 1 | 21 | 21.9 |  |  |  | 81.4 | 69.9 | 94.8 | 2.7 |  |
|  |  | **AA** | 2 | 1 | 0.5 | 0.43 | 0.51 |  | 96.6 | 47.7 | 195.8 | 21.9 | 0.82 |
|  |  | **GA+AA** |  | 23 |  |  |  |  | 82.1 | 70.7 | 95.2 | 3.5 | 0.66 |
|  |  |  |  |  |  |  |  |  |  |  |  |  |  |
| ***LIG1*** | **rs3730947** |  |  |  |  |  |  |  |  |  |  |  |  |
|  |  | **Missing** | 0 | 0 |  |  |  |  |  |  |  |  |  |
|  |  | **GG** | 0 | 245 |  |  |  |  | 79.4 | 75.9 | 83.0 | - | NA |
|  |  |  |  |  |  |  |  |  |  |  |  |  |  |
| ***LIG1*** | **rs3731037**^d^ |  |  |  |  |  |  |  |  |  |  |  |  |
|  |  | **Missing** | 0 | 1 |  |  |  |  |  |  |  |  |  |
|  |  | **CC** | 0 | 190 | 188.2 |  |  |  | 78.5 | 74.6 | 82.6 | - |  |
|  |  | **CT** | 1 | 45 | 48.7 |  |  |  | 83.2 | 75.3 | 91.9 | 6.0 |  |
|  |  | **TT** | 2 | 5 | 3.2 | 1.38 | 0.24 |  | 70.6 | 51.8 | 96.3 | -10.0 | 0.45 |
|  |  | **CT+TT** |  | 50 |  |  |  |  | 81.9 | 74.5 | 90.0 | 4.3 | 0.44 |
|  |  |  |  |  |  |  |  |  |  |  |  |  |  |
| ***LIG1*** | **rs411073** |  |  |  |  |  |  |  |  |  |  |  |  |
|  |  | **Missing** | 1 | 6 |  |  |  |  |  |  |  |  |  |
|  |  | **CC** | 0 | 78 | 74.6 |  |  |  | 78.2 | 72.2 | 84.7 | - |  |
|  |  | **CT** | 1 | 111 | 117.9 |  |  |  | 81.5 | 76.2 | 87.1 | 4.2 |  |
|  |  | **TT** | 2 | 50 | 46.6 | 0.81 | 0.37 |  | 75.8 | 68.6 | 83.8 | -3.0 | 0.46 |
|  |  |  |  |  |  |  |  |  |  |  |  |  |  |
| ***LIG1*** | **rs3730908**^d^ |  |  |  |  |  |  |  |  |  |  |  |  |
|  |  | **Missing** | 0 | 2 |  |  |  |  |  |  |  |  |  |
|  |  | **CC** | 0 | 224 | 224.4 |  |  |  | 81.8 | 78.1 | 85.7 | - |  |
|  |  | **CT** | 1 | 19 | 18.3 | 0.03 | 0.86 |  | 80.0 | 68.5 | 93.3 | -2.3 | 0.90 |
|  |  |  |  |  |  |  |  |  |  |  |  |  |  |
| ***LIG1*** | **rs20579**^d^ |  |  |  |  |  |  |  |  |  |  |  |  |
|  |  | **Missing** | 0 | 1 |  |  |  |  |  |  |  |  |  |
|  |  | **CC** | 0 | 183 | 184.2 |  |  |  | 79.2 | 75.3 | 83.4 | - |  |
|  |  | **CT** | 1 | 58 | 55.6 |  |  |  | 80.5 | 73.5 | 88.2 | 1.7 |  |
|  |  | **TT** | 2 | 3 | 4.2 | 0.45 | 0.50 |  | 60.7 | 40.7 | 90.6 | -23.4 | 0.40 |
|  |  | **CT+TT** |  | 61 |  |  |  |  | 79.4 | 72.6 | 86.8 | 0.2 | 0.96 |
|  |  |  |  |  |  |  |  |  |  |  |  |  |  |
| ***LIG1*** | **rs3730881** |  |  |  |  |  |  |  |  |  |  |  |  |
|  |  | **Missing** | 0 | 0 |  |  |  |  |  |  |  |  |  |
|  |  | **CC** | 0 | 241 | 241.0 |  |  |  | 79.0 | 75.5 | 82.6 | - |  |
|  |  | **CT** | 1 | 4 | 4.0 | 0.00 | 0.99 |  | 106.4 | 75.0 | 150.9 | 34.7 | 0.10 |
|  |  |  |  |  |  |  |  |  |  |  |  |  |  |
| ***LIG1*** | **rs3730914**^d^ |  |  |  |  |  |  |  |  |  |  |  |  |
|  |  | **Missing** | 0 | 3 |  |  |  |  |  |  |  |  |  |
|  |  | **CC** | 0 | 165 | 166.9 |  |  |  | 78.4 | 74.3 | 82.8 | - |  |
|  |  | **CT** | 1 | 72 | 68.1 |  |  |  | 82.0 | 75.5 | 89.0 | 4.5 |  |
|  |  | **TT** | 2 | 5 | 6.9 | 0.79 | 0.37 |  | 68.1 | 49.8 | 92.9 | -13.2 | 0.42 |
|  |  | **CT+TT** |  | 77 |  |  |  |  | 81.0 | 74.8 | 87.8 | 3.3 | 0.51 |
|  |  |  |  |  |  |  |  |  |  |  |  |  |  |
| ***LIG1*** | **rs3731003** |  |  |  |  |  |  |  |  |  |  |  |  |
|  |  | **Missing** | 0 | 3 |  |  |  |  |  |  |  |  |  |
|  |  | **CC** | 0 | 242 |  |  |  |  | 79.2 | 75.7 | 82.8 | - | NA |
|  |  |  |  |  |  |  |  |  |  |  |  |  |  |
| ***LIG1*** | **rs3730837**^d^ |  |  |  |  |  |  |  |  |  |  |  |  |
|  |  | **Missing** | 0 | 6 |  |  |  |  |  |  |  |  |  |
|  |  | **AA** | 0 | 183 | 185.4 |  |  |  | 79.3 | 75.3 | 83.5 | - |  |
|  |  | **AG** | 1 | 55 | 50.2 |  |  |  | 78.2 | 71.2 | 85.8 | -1.5 |  |
|  |  | **GG** | 2 | 1 | 3.4 | 2.18 | 0.14 |  | 46.3 | 21.7 | 98.8 | -41.7 | 0.37 |
|  |  | **AG+GG** |  | 56 |  |  |  |  | 77.4 | 70.6 | 84.9 | -2.4 | 0.65 |
|  |  |  |  |  |  |  |  |  |  |  |  |  |  |
| ***LIG1*** | **rs274862** |  |  |  |  |  |  |  |  |  |  |  |  |
|  |  | **Missing** | 1 | 6 |  |  |  |  |  |  |  |  |  |
|  |  | **TT** | 0 | 87 | 85.0 |  |  |  | 79.6 | 73.8 | 85.8 | - |  |
|  |  | **TC** | 1 | 111 | 115.1 |  |  |  | 77.3 | 72.3 | 82.5 | -2.9 |  |
|  |  | **CC** | 2 | 41 | 39.0 | 0.30 | 0.58 |  | 81.6 | 73.2 | 91.1 | 2.6 | 0.67 |
|  |  |  |  |  |  |  |  |  |  |  |  |  |  |
| ***LIG1*** | **rs3730912**^d^ |  |  |  |  |  |  |  |  |  |  |  |  |
|  |  | **Missing** | 0 | 1 |  |  |  |  |  |  |  |  |  |
|  |  | **CC** | 0 | 187 | 185.9 |  |  |  | 79.2 | 75.3 | 83.4 | - |  |
|  |  | **CA** | 1 | 52 | 54.1 |  |  |  | 81.9 | 74.4 | 90.2 | 3.4 |  |
|  |  | **AA** | 2 | 5 | 3.9 | 0.38 | 0.54 |  | 70.6 | 51.8 | 96.1 | -11.0 | 0.62 |
|  |  | **CA+AA** |  | 57 |  |  |  |  | 80.8 | 73.8 | 88.6 | 2.0 | 0.71 |
|  |  |  |  |  |  |  |  |  |  |  |  |  |  |
| ***LIG1*** | **rs20580** |  |  |  |  |  |  |  |  |  |  |  |  |
|  |  | **Missing** | 1 | 5 |  |  |  |  |  |  |  |  |  |
|  |  | **CC** | 0 | 63 | 58.0 |  |  |  | 79.7 | 72.9 | 87.1 | - |  |
|  |  | **CA** | 1 | 110 | 120.0 |  |  |  | 78.8 | 73.7 | 84.2 | -1.1 |  |
|  |  | **AA** | 2 | 67 | 62.0 | 1.66 | 0.20 |  | 79.6 | 73.1 | 86.7 | -0.1 | 0.97 |
|  |  |  |  |  |  |  |  |  |  |  |  |  |  |
| ***LIG3*** | **rs3135974**^d^ |  |  |  |  |  |  |  |  |  |  |  |  |
|  |  | **Missing** | 0 | 1 |  |  |  |  |  |  |  |  |  |
|  |  | **GG** | 0 | 202 | 202.0 |  |  |  | 79.0 | 75.2 | 83.0 | - |  |
|  |  | **GA** | 1 | 40 | 40.0 |  |  |  | 80.9 | 72.4 | 90.3 | 2.4 |  |
|  |  | **AA** | 2 | 2 | 2.0 | 0.00 | 0.99 |  | 83.7 | 51.0 | 137.4 | 6.0 | 0.91 |
|  |  | **GA+AA** |  | 42 |  |  |  |  | 81.0 | 72.8 | 90.2 | 2.5 | 0.68 |
|  |  |  |  |  |  |  |  |  |  |  |  |  |  |
| ***LIG3*** | **rs3135998** |  |  |  |  |  |  |  |  |  |  |  |  |
|  |  | **Missing** | 1 | 0 |  |  |  |  |  |  |  |  |  |
|  |  | **GG** | 0 | 78 | 76.1 |  |  |  | 80.0 | 74.0 | 86.6 | - |  |
|  |  | **GA** | 1 | 117 | 120.9 |  |  |  | 78.2 | 73.3 | 83.4 | -2.3 |  |
|  |  | **AA** | 2 | 50 | 48.1 | 0.25 | 0.61 |  | 81.1 | 73.5 | 89.5 | 1.4 | 0.98 |
|  |  |  |  |  |  |  |  |  |  |  |  |  |  |
| ***LIG3*** | **rs3135989**^d^ |  |  |  |  |  |  |  |  |  |  |  |  |
|  |  | **Missing** | 0 | 2 |  |  |  |  |  |  |  |  |  |
|  |  | **TT** | 0 | 216 | 215.8 |  |  |  | 81.6 | 77.9 | 85.5 | - |  |
|  |  | **TG** | 1 | 26 | 26.4 |  |  |  | 82.5 | 72.2 | 94.4 | 1.1 |  |
|  |  | **GG** | 2 | 1 | 0.8 | 0.05 | 0.82 |  | 63.7 | 32.4 | 125.0 | -22.0 | 0.61 |
|  |  | **TG+GG** |  | 27 |  |  |  |  | 81.7 | 71.7 | 93.2 | 0.1 | 0.62 |
|  |  |  |  |  |  |  |  |  |  |  |  |  |  |
| ***LIG3*** | **rs3135967** |  |  |  |  |  |  |  |  |  |  |  |  |
|  |  | **Missing** | 1 | 4 |  |  |  |  |  |  |  |  |  |
|  |  | **AA** | 0 | 79 | 76.7 |  |  |  | 79.5 | 73.6 | 85.8 | - |  |
|  |  | **AG** | 1 | 114 | 118.5 |  |  |  | 78.9 | 74.0 | 84.1 | -0.7 |  |
|  |  | **GG** | 2 | 48 | 45.7 | 0.35 | 0.55 |  | 78.4 | 71.1 | 86.6 | -1.3 | 0.98 |
|  |  |  |  |  |  |  |  |  |  |  |  |  |  |
| ***LIG3*** | **rs2074516**^d^ |  |  |  |  |  |  |  |  |  |  |  |  |
|  |  | **Missing** | 0 | 1 |  |  |  |  |  |  |  |  |  |
|  |  | **GG** | 0 | 200 | 200.2 |  |  |  | 81.5 | 77.6 | 85.5 | - |  |
|  |  | **GC** | 1 | 42 | 41.7 |  |  |  | 83.7 | 75.5 | 92.8 | 2.7 |  |
|  |  | **CC** | 2 | 2 | 2.2 | 0.02 | 0.90 |  | 77.8 | 39.6 | 152.7 | -4.5 | 0.93 |
|  |  | **GC+CC** |  | 44 |  |  |  |  | 83.6 | 75.5 | 92.6 | 2.6 | 0.72 |
|  |  |  |  |  |  |  |  |  |  |  |  |  |  |
| ***MBD4*** | **rs3138360**^d^ |  |  |  |  |  |  |  |  |  |  |  |  |
|  |  | **Missing** | 0 | 1 |  |  |  |  |  |  |  |  |  |
|  |  | **GG** | 0 | 220 | 220.6 |  |  |  | 81.7 | 78.0 | 85.6 | - |  |
|  |  | **GA** | 1 | 24 | 22.8 | 0.06 | 0.80 |  | 82.5 | 72.3 | 94.2 | 1.0 | 0.60 |
|  |  |  |  |  |  |  |  |  |  |  |  |  |  |
| ***MBD4*** | **rs10342**^d^ |  |  |  |  |  |  |  |  |  |  |  |  |
|  |  | **Missing** | 0 | 1 |  |  |  |  |  |  |  |  |  |
|  |  | **GG** | 0 | 200 | 198.4 |  |  |  | 80.5 | 76.6 | 84.6 | - |  |
|  |  | **GA** | 1 | 40 | 43.3 |  |  |  | 74.5 | 66.7 | 83.2 | -7.4 |  |
|  |  | **AA** | 2 | 4 | 2.4 | 1.40 | 0.24 |  | 85.3 | 60.3 | 120.7 | 6.0 | 0.42 |
|  |  | **GA+AA** |  | 44 |  |  |  |  | 75.4 | 67.9 | 83.8 | -6.3 | 0.28 |
|  |  |  |  |  |  |  |  |  |  |  |  |  |  |
| ***MBD4*** | **rs2005618**^d^ |  |  |  |  |  |  |  |  |  |  |  |  |
|  |  | **Missing** | 0 | 0 |  |  |  |  |  |  |  |  |  |
|  |  | **TT** | 0 | 193 | 192.2 |  |  |  | 82.4 | 78.4 | 86.5 | - |  |
|  |  | **TC** | 1 | 48 | 49.6 |  |  |  | 79.0 | 71.6 | 87.2 | -4.0 |  |
|  |  | **CC** | 2 | 4 | 3.2 | 0.25 | 0.61 |  | 88.4 | 63.1 | 123.7 | 7.3 | 0.62 |
|  |  | **TC+CC** |  | 52 |  |  |  |  | 79.7 | 72.5 | 87.6 | -3.2 | 0.45 |
|  |  |  |  |  |  |  |  |  |  |  |  |  |  |
| ***MBD4*** | **rs2311394**^d^ |  |  |  |  |  |  |  |  |  |  |  |  |
|  |  | **Missing** | 0 | 5 |  |  |  |  |  |  |  |  |  |
|  |  | **TT** | 0 | 195 | 194.4 |  |  |  | 80.5 | 76.6 | 84.6 | - |  |
|  |  | **TC** | 1 | 42 | 43.2 |  |  |  | 76.8 | 69.0 | 85.5 | -4.6 |  |
|  |  | **CC** | 2 | 3 | 2.4 | 0.19 | 0.67 |  | 75.8 | 50.9 | 112.9 | -5.8 | 0.72 |
|  |  | **TC+CC** |  | 45 |  |  |  |  | 76.8 | 69.2 | 85.1 | -4.7 | 0.42 |
|  |  |  |  |  |  |  |  |  |  |  |  |  |  |
| ***MBD4*** | **rs2307293** |  |  |  |  |  |  |  |  |  |  |  |  |
|  |  | **Missing** | 0 | 4 |  |  |  |  |  |  |  |  |  |
|  |  | **GG** | 0 | 238 | 238.0 |  |  |  | 79.2 | 75.7 | 82.9 | - |  |
|  |  | **GC** | 1 | 3 | 3.0 | 0.00 | 0.99 |  | 76.4 | 51.3 | 113.9 | -3.5 | 0.86 |
|  |  |  |  |  |  |  |  |  |  |  |  |  |  |
| ***MBD4*** | **rs3138326**^d^ |  |  |  |  |  |  |  |  |  |  |  |  |
|  |  | **Missing** | 0 | 10 |  |  |  |  |  |  |  |  |  |
|  |  | **AA** | 0 | 190 | 188.6 |  |  |  | 79.7 | 75.8 | 83.8 | - |  |
|  |  | **AT** | 1 | 41 | 43.9 |  |  |  | 76.6 | 68.8 | 85.4 | -3.8 |  |
|  |  | **TT** | 2 | 4 | 2.6 | 1.02 | 0.31 |  | 85.4 | 60.5 | 120.4 | 7.1 | 0.74 |
|  |  | **AT+TT** |  | 45 |  |  |  |  | 77.4 | 69.8 | 85.8 | -2.9 | 0.62 |
|  |  |  |  |  |  |  |  |  |  |  |  |  |  |
| ***MPG*** | **rs3176415** |  |  |  |  |  |  |  |  |  |  |  |  |
|  |  | **Missing** | 1 | 5 |  |  |  |  |  |  |  |  |  |
|  |  | **GG** | 0 | 75 | 73.7 |  |  |  | 75.8 | 70.0 | 82.2 | - |  |
|  |  | **GA** | 1 | 116 | 118.6 |  |  |  | 82.4 | 77.2 | 87.8 | 8.6 |  |
|  |  | **AA** | 2 | 49 | 47.7 | 0.11 | 0.73 |  | 77.3 | 70.0 | 85.4 | 1.9 | 0.25 |
|  |  |  |  |  |  |  |  |  |  |  |  |  |  |
| ***MPG*** | **rs2541622**^d^ |  |  |  |  |  |  |  |  |  |  |  |  |
|  |  | **Missing** | 0 | 3 |  |  |  |  |  |  |  |  |  |
|  |  | **CC** | 0 | 165 | 165.3 |  |  |  | 78.6 | 74.5 | 83.0 | - |  |
|  |  | **CT** | 1 | 70 | 69.4 |  |  |  | 80.9 | 74.5 | 88.0 | 3.0 |  |
|  |  | **TT** | 2 | 7 | 7.3 | 0.02 | 0.90 |  | 78.1 | 59.9 | 101.9 | -0.6 | 0.84 |
|  |  | **CT+TT** |  | 77 |  |  |  |  | 80.7 | 74.5 | 87.4 | 2.7 | 0.59 |
|  |  |  |  |  |  |  |  |  |  |  |  |  |  |
| ***MPG*** | **rs3176424** |  |  |  |  |  |  |  |  |  |  |  |  |
|  |  | **Missing** | 0 | 0 |  |  |  |  |  |  |  |  |  |
|  |  | **AA** | 0 | 238 | 238.1 |  |  |  | 79.5 | 76.0 | 83.2 | - |  |
|  |  | **AG** | 1 | 7 | 6.9 | 0.00 | 0.97 |  | 75.2 | 57.8 | 97.7 | -5.4 | 0.68 |
|  |  |  |  |  |  |  |  |  |  |  |  |  |  |
| ***MUTYH*** | **rs3219476** |  |  |  |  |  |  |  |  |  |  |  |  |
|  |  | **Missing** | 1 | 0 |  |  |  |  |  |  |  |  |  |
|  |  | **GG** | 0 | 91 | 95.5 |  |  |  | 79.9 | 74.4 | 85.9 | - |  |
|  |  | **GT** | 1 | 124 | 114.9 |  |  |  | 75.6 | 71.1 | 80.4 | -5.4 |  |
|  |  | **TT** | 2 | 30 | 34.5 | 1.53 | 0.22 |  | 95.3 | 84.0 | 108.2 | 19.2 | 0.005 |
|  |  |  |  |  |  |  |  |  |  |  |  |  |  |
| ***MUTYH*** | **rs3219484**^d^ |  |  |  |  |  |  |  |  |  |  |  |  |
|  |  | **Missing** | 0 | 1 |  |  |  |  |  |  |  |  |  |
|  |  | **GG** | 0 | 211 | 211.2 |  |  |  | 82.9 | 79.1 | 86.9 | - |  |
|  |  | **GA** | 1 | 32 | 31.6 |  |  |  | 77.2 | 68.5 | 86.9 | -6.9 |  |
|  |  | **AA** | 2 | 1 | 1.2 | 0.03 | 0.86 |  | 52.9 | 33.0 | 84.7 | -36.2 | 0.37 |
|  |  | **GA+AA** |  | 33 |  |  |  |  | 75.4 | 67.2 | 84.7 | -9.1 | 0.42 |
|  |  |  |  |  |  |  |  |  |  |  |  |  |  |
| ***MUTYH*** | **rs3219494** |  |  |  |  |  |  |  |  |  |  |  |  |
|  |  | **Missing** | 0 | 0 |  |  |  |  |  |  |  |  |  |
|  |  | **GG** | 0 | 245 |  |  |  |  | 79.4 | 75.9 | 83.0 | - | NA |
|  |  |  |  |  |  |  |  |  |  |  |  |  |  |
| ***MUTYH*** | **rs3219463** |  |  |  |  |  |  |  |  |  |  |  |  |
|  |  | **Missing** | 0 | 3 |  |  |  |  |  |  |  |  |  |
|  |  | **GG** | 0 | 130 | 133.9 |  |  |  | 79.1 | 74.4 | 84.0 | - |  |
|  |  | **GA** | 1 | 100 | 92.2 |  |  |  | 78.7 | 73.4 | 84.3 | -0.5 |  |
|  |  | **AA** | 2 | 12 | 15.9 | 1.72 | 0.19 |  | 93.3 | 76.2 | 114.2 | 18.0 | 0.28 |
|  |  |  |  |  |  |  |  |  |  |  |  |  |  |
| ***MUTYH*** | **rs3219489** |  |  |  |  |  |  |  |  |  |  |  |  |
|  |  | **Missing** | 0 | 0 |  |  |  |  |  |  |  |  |  |
|  |  | **GG** | 0 | 131 | 133.7 |  |  |  | 78.8 | 74.2 | 83.8 | - |  |
|  |  | **GC** | 1 | 100 | 94.6 |  |  |  | 78.1 | 72.9 | 83.7 | -0.9 |  |
|  |  | **CC** | 2 | 14 | 16.7 | 0.81 | 0.37 |  | 95.0 | 78.8 | 114.5 | 20.5 | 0.15 |
|  |  |  |  |  |  |  |  |  |  |  |  |  |  |
| ***MUTYH*** | **rs3219493**^d^ |  |  |  |  |  |  |  |  |  |  |  |  |
|  |  | **Missing** | 0 | 6 |  |  |  |  |  |  |  |  |  |
|  |  | **GG** | 0 | 186 | 188.1 |  |  |  | 78.3 | 74.5 | 82.4 | - |  |
|  |  | **GC** | 1 | 52 | 47.9 |  |  |  | 80.3 | 73.0 | 88.4 | 2.5 | 0.58 |
|  |  | **CC** | 2 | 1 | 3.1 | 1.75 | 0.19 |  | 51.0 | 25.5 | 102.0 | -34.9 | 0.42 |
|  |  | **GC+CC** |  | 53 |  |  |  |  | 79.6 | 72.4 | 87.6 | 1.6 | 0.77 |
|  |  |  |  |  |  |  |  |  |  |  |  |  |  |
| ***OGG1*** | **rs125701^d^** |  |  |  |  |  |  |  |  |  |  |  |  |
|  |  | **Missing** | 0 | 2 |  |  |  |  |  |  |  |  |  |
|  |  | **GG** | 0 | 173 | 171.3 |  |  |  | 82.7 | 78.5 | 87.1 | - |  |
|  |  | **GA** | 1 | 62 | 65.5 |  |  |  | 72.1 | 66.1 | 78.6 | -12.8 |  |
|  |  | **AA** | 2 | 8 | 6.3 | 0.69 | 0.41 |  | 63.4 | 49.8 | 80.7 | -23.3 | 0.006 |
|  |  | **GA+AA** |  | 70 |  |  |  |  | 71.0 | 65.5 | 77.1 | -14.1 | 0.002 |
|  |  |  |  |  |  |  |  |  |  |  |  |  |  |
| ***OGG1*** | **rs1805373** |  |  |  |  |  |  |  |  |  |  |  |  |
|  |  | **Missing** | 0 | 0 |  |  |  |  |  |  |  |  |  |
|  |  | **GG** | 0 | 245 |  |  |  |  | 79.4 | 75.9 | 83.0 | - | NA |
|  |  |  |  |  |  |  |  |  |  |  |  |  |  |
| ***OGG1*** | **rs2072668** |  |  |  |  |  |  |  |  |  |  |  |  |
|  |  | **Missing** | 0 | 1 |  |  |  |  |  |  |  |  |  |
|  |  | **CC** | 0 | 145 | 146.4 |  |  |  | 76.7 | 72.4 | 81.2 | - |  |
|  |  | **CG** | 1 | 88 | 85.2 |  |  |  | 82.8 | 76.9 | 89.2 | 8.0 |  |
|  |  | **GG** | 2 | 11 | 12.4 | 0.26 | 0.61 |  | 89.8 | 72.8 | 110.7 | 17.1 | 0.14 |
|  |  |  |  |  |  |  |  |  |  |  |  |  |  |
| ***OGG1*** | **rs3219008** |  |  |  |  |  |  |  |  |  |  |  |  |
|  |  | **Missing** | 0 | 2 |  |  |  |  |  |  |  |  |  |
|  |  | **AA** | 0 | 152 | 148.6 |  |  |  | 76.5 | 72.4 | 80.9 | - |  |
|  |  | **AG** | 1 | 76 | 82.9 |  |  |  | 81.3 | 75.1 | 87.9 | 6.2 |  |
|  |  | **GG** | 2 | 15 | 11.6 | 1.67 | 0.20 |  | 96.1 | 80.5 | 114.7 | 25.6 | 0.04 |
|  |  |  |  |  |  |  |  |  |  |  |  |  |  |
| ***OGG1*** | **rs159153** |  |  |  |  |  |  |  |  |  |  |  |  |
|  |  | **Missing** | 0 | 6 |  |  |  |  |  |  |  |  |  |
|  |  | **TT** | 0 | 117 | 111.2 |  |  |  | 82.9 | 77.8 | 88.4 | - |  |
|  |  | **TC** | 1 | 92 | 103.7 |  |  |  | 78.7 | 73.3 | 84.6 | -5.1 |  |
|  |  | **CC** | 2 | 30 | 24.2 | 3.03 | 0.08 |  | 68.4 | 60.3 | 77.6 | -17.5 | 0.03 |
|  |  |  |  |  |  |  |  |  |  |  |  |  |  |
| ***OGG1*** | **rs293795** |  |  |  |  |  |  |  |  |  |  |  |  |
|  |  | **Missing** | 0 | 0 |  |  |  |  |  |  |  |  |  |
|  |  | **TT** | 0 | 164 | 162.5 |  |  |  | 83.3 | 78.9 | 87.8 | - |  |
|  |  | **TC** | 1 | 71 | 74.1 |  |  |  | 73.3 | 67.6 | 79.5 | -11.9 |  |
|  |  | **CC** | 2 | 10 | 8.5 | 0.43 | 0.51 |  | 63.8 | 51.4 | 79.1 | -23.4 | 0.005 |
|  |  |  |  |  |  |  |  |  |  |  |  |  |  |
| ***PNKP*** | **rs3739206** |  |  |  |  |  |  |  |  |  |  |  |  |
|  |  | **Missing** | 0 | 1 |  |  |  |  |  |  |  |  |  |
|  |  | **TT** | 0 | 243 | 242.0 |  |  |  | 79.4 | 76.0 | 83.1 | - |  |
|  |  | **GG** | 2 | 1 | 0.0 | 242.01 | < 0.001 |  | 64.0 | 31.9 | 128.5 | -19.4 | 0.54 |
|  |  |  |  |  |  |  |  |  |  |  |  |  |  |
| ***PNKP*** | **rs2257103** |  |  |  |  |  |  |  |  |  |  |  |  |
|  |  | **Missing** | 1 | 7 |  |  |  |  |  |  |  |  |  |
|  |  | **CC** | 0 | 93 | 84.1 |  |  |  | 83.0 | 77.3 | 89.2 | - |  |
|  |  | **CT** | 1 | 97 | 114.7 |  |  |  | 78.4 | 73.0 | 84.1 | -5.6 |  |
|  |  | **TT** | 2 | 48 | 39.1 | 5.69 | 0.02 |  | 75.1 | 68.0 | 83.0 | -9.5 | 0.25 |
|  |  |  |  |  |  |  |  |  |  |  |  |  |  |
| ***PNKP*** | **rs3739186** |  |  |  |  |  |  |  |  |  |  |  |  |
|  |  | **Missing** | 0 | 1 |  |  |  |  |  |  |  |  |  |
|  |  | **TT** | 0 | 244 |  |  |  |  | 79.2 | 75.8 | 82.8 | - | NA |
|  |  |  |  |  |  |  |  |  |  |  |  |  |  |
| ***POLB*** | **rs2979896**^d^ |  |  |  |  |  |  |  |  |  |  |  |  |
|  |  | **Missing** | 0 | 1 |  |  |  |  |  |  |  |  |  |
|  |  | **TT** | 0 | 215 | 215.9 |  |  |  | 78.9 | 75.2 | 82.8 | - |  |
|  |  | **TG** | 1 | 29 | 27.3 | 0.11 | 0.74 |  | 82.4 | 72.4 | 93.8 | 4.5 | 0.53 |
|  |  |  |  |  |  |  |  |  |  |  |  |  |  |
| ***POLB*** | **rs3136811**^d^ |  |  |  |  |  |  |  |  |  |  |  |  |
|  |  | **Missing** | 0 | 2 |  |  |  |  |  |  |  |  |  |
|  |  | **CC** | 0 | 215 | 215.9 |  |  |  | 78.9 | 75.3 | 82.8 | - |  |
|  |  | **CG** | 1 | 30 | 28.2 | 0.12 | 0.73 |  | 82.6 | 72.7 | 93.7 | 4.6 | 0.51 |
|  |  |  |  |  |  |  |  |  |  |  |  |  |  |
| ***POLB*** | **rs3136797** |  |  |  |  |  |  |  |  |  |  |  |  |
|  |  | **Missing** | 0 | 8 |  |  |  |  |  |  |  |  |  |
|  |  | **CC** | 0 | 232 | 232.0 |  |  |  | 79.4 | 75.9 | 83.2 | - |  |
|  |  | **CG** | 1 | 5 | 4.9 | 0.00 | 0.98 |  | 79.1 | 58.0 | 108.0 | -0.4 | 0.98 |
|  |  |  |  |  |  |  |  |  |  |  |  |  |  |
| ***SMUG1*** | **rs2233920** |  |  |  |  |  |  |  |  |  |  |  |  |
|  |  | **Missing** | 0 | 1 |  |  |  |  |  |  |  |  |  |
|  |  | **GG** | 0 | 244 |  |  |  |  | 79.6 | 76.2 | 83.2 |  | NA |
|  |  |  |  |  |  |  |  |  |  |  |  |  |  |
| ***SMUG1*** | **rs3136386**^d^ |  |  |  |  |  |  |  |  |  |  |  |  |
|  |  | **Missing** | 0 | 2 |  |  |  |  |  |  |  |  |  |
|  |  | **CC** | 0 | 225 | 225.3 |  |  |  | 79.2 | 75.6 | 83.0 | - |  |
|  |  | **CG** | 1 | 18 | 17.3 | 0.03 | 0.87 |  | 79.6 | 67.5 | 93.9 | 0.5 | 0.96 |
|  |  |  |  |  |  |  |  |  |  |  |  |  |  |
| ***SMUG1*** | **rs971** |  |  |  |  |  |  |  |  |  |  |  |  |
|  |  | **Missing** | 0 | 2 |  |  |  |  |  |  |  |  |  |
|  |  | **CC** | 0 | 103 | 100.1 |  |  |  | 82.2 | 76.8 | 88.0 | - |  |
|  |  | **CT** | 1 | 106 | 111.7 |  |  |  | 77.8 | 72.7 | 83.2 | -5.3 |  |
|  |  | **TT** | 2 | 34 | 31.1 | 0.63 | 0.43 |  | 74.9 | 66.4 | 84.5 | -8.8 | 0.33 |
|  |  |  |  |  |  |  |  |  |  |  |  |  |  |
| ***SMUG1*** | **rs2279402** |  |  |  |  |  |  |  |  |  |  |  |  |
|  |  | **Missing** | 1 | 1 |  |  |  |  |  |  |  |  |  |
|  |  | **CC** | 0 | 74 | 71.4 |  |  |  | 83.1 | 76.7 | 90.1 | - |  |
|  |  | **CT** | 1 | 116 | 121.2 |  |  |  | 77.8 | 72.9 | 83.0 | -6.4 |  |
|  |  | **TT** | 2 | 54 | 51.4 | 0.45 | 0.50 |  | 77.7 | 70.7 | 85.5 | -6.5 | 0.40 |
|  |  |  |  |  |  |  |  |  |  |  |  |  |  |
| ***TDG*** | **rs3829301**^d^ |  |  |  |  |  |  |  |  |  |  |  |  |
|  |  | **Missing** | 0 | 0 |  |  |  |  |  |  |  |  |  |
|  |  | **AA** | 0 | 220 | 218.7 |  |  |  | 79.1 | 75.5 | 82.9 | - |  |
|  |  | **AC** | 1 | 23 | 25.5 |  |  |  | 83.7 | 72.4 | 96.7 | 5.7 |  |
|  |  | **CC** | 2 | 2 | 0.7 | 2.38 | 0.12 |  | 58.9 | 36.1 | 96.2 | -25.5 | 0.38 |
|  |  | **AC+CC** |  | 25 |  |  |  |  | 81.3 | 70.7 | 93.5 | 2.8 | 0.71 |
|  |  |  |  |  |  |  |  |  |  |  |  |  |  |
| ***TDG*** | **rs4135113** |  |  |  |  |  |  |  |  |  |  |  |  |
|  |  | **Missing** | 0 | 3 |  |  |  |  |  |  |  |  |  |
|  |  | **GG** | 0 | 231 | 231.1 |  |  |  | 79.0 | 75.4 | 82.6 | - |  |
|  |  | **GA** | 1 | 11 | 10.8 | 0.01 | 0.94 |  | 80.7 | 65.5 | 99.3 | 2.2 | 0.84 |
|  |  |  |  |  |  |  |  |  |  |  |  |  |  |
| ***TDG*** | **rs2629768**^d^ |  |  |  |  |  |  |  |  |  |  |  |  |
|  |  | **Missing** | 0 | 10 |  |  |  |  |  |  |  |  |  |
|  |  | **GG** | 0 | 177 | 175.4 |  |  |  | 80.3 | 76.2 | 84.6 | - |  |
|  |  | **GA** | 1 | 52 | 55.3 |  |  |  | 75.2 | 68.3 | 82.8 | -6.3 |  |
|  |  | **AA** | 2 | 6 | 4.4 | 0.83 | 0.36 |  | 61.6 | 46.4 | 81.8 | -23.2 | 0.12 |
|  |  | **GA+AA** |  | 58 |  |  |  |  | 73.7 | 67.3 | 80.7 | -8.2 | 0.11 |
|  |  |  |  |  |  |  |  |  |  |  |  |  |  |
| ***TDG*** | **rs4135064**^d^ |  |  |  |  |  |  |  |  |  |  |  |  |
|  |  | **Missing** | 0 | 1 |  |  |  |  |  |  |  |  |  |
|  |  | **CC** | 0 | 203 | 202.9 |  |  |  | 79.0 | 75.2 | 83.0 | - |  |
|  |  | **CT** | 1 | 39 | 39.2 |  |  |  | 80.6 | 72.1 | 90.1 | 2.0 |  |
|  |  | **TT** | 2 | 2 | 1.9 | 0.01 | 0.93 |  | 95.5 | 58.3 | 156.6 | 20.8 | 0.72 |
|  |  | **CT+TT** |  | 41 |  |  |  |  | 81.3 | 72.9 | 90.6 | 2.9 | 0.65 |
|  |  |  |  |  |  |  |  |  |  |  |  |  |  |
| ***TDG*** | **rs322107**^d^ |  |  |  |  |  |  |  |  |  |  |  |  |
|  |  | **Missing** | 0 | 1 |  |  |  |  |  |  |  |  |  |
|  |  | **CC** | 0 | 182 | 180.7 |  |  |  | 81.1 | 77.1 | 85.4 | - |  |
|  |  | **CT** | 1 | 56 | 58.5 |  |  |  | 75.7 | 69.0 | 83.0 | -6.7 |  |
|  |  | **TT** | 2 | 6 | 4.7 | 0.45 | 0.50 |  | 61.7 | 46.5 | 81.9 | -24.0 | 0.09 |
|  |  | **CT+TT** |  | 62 |  |  |  |  | 74.3 | 68.0 | 81.1 | -8.5 | 0.09 |
|  |  |  |  |  |  |  |  |  |  |  |  |  |  |
| ***TDG*** | **rs4135061** |  |  |  |  |  |  |  |  |  |  |  |  |
|  |  | **Missing** | 0 | 2 |  |  |  |  |  |  |  |  |  |
|  |  | **AA** | 0 | 138 | 134.8 |  |  |  | 82.5 | 77.8 | 87.5 | - |  |
|  |  | **AG** | 1 | 86 | 92.4 |  |  |  | 77.7 | 72.2 | 83.7 | -5.7 |  |
|  |  | **GG** | 2 | 19 | 15.8 | 1.15 | 0.28 |  | 66.6 | 56.8 | 78.0 | -19.3 | 0.04 |
|  |  |  |  |  |  |  |  |  |  |  |  |  |  |
| ***TDG*** | **rs4135081** |  |  |  |  |  |  |  |  |  |  |  |  |
|  |  | **Missing** | 1 | 0 |  |  |  |  |  |  |  |  |  |
|  |  | **AA** | 0 | 79 | 78.3 |  |  |  | 76.8 | 71.0 | 83.1 | - |  |
|  |  | **AG** | 1 | 119 | 120.4 |  |  |  | 80.8 | 75.8 | 86.1 | 5.1 |  |
|  |  | **GG** | 2 | 47 | 46.3 | 0.03 | 0.85 |  | 80.2 | 72.4 | 88.9 | 4.4 | 0.60 |
|  |  |  |  |  |  |  |  |  |  |  |  |  |  |
| ***TDG*** | **rs322109**^d^ |  |  |  |  |  |  |  |  |  |  |  |  |
|  |  | **Missing** | 0 | 15 |  |  |  |  |  |  |  |  |  |
|  |  | **AA** | 0 | 191 | 186.3 |  |  |  | 80.5 | 76.4 | 84.7 | - |  |
|  |  | **AG** | 1 | 32 | 41.4 |  |  |  | 77.2 | 68.2 | 87.4 | -4.1 |  |
|  |  | **GG** | 2 | 7 | 2.3 | 11.86 | < 0.001 |  | 69.0 | 52.9 | 90.0 | -14.2 | 0.47 |
|  |  | **AG+GG** |  | 39 |  |  |  |  | 75.7 | 67.6 | 84.7 | -6.0 | 0.33 |
|  |  |  |  |  |  |  |  |  |  |  |  |  |  |
| ***TDG*** | **rs4135093** |  |  |  |  |  |  |  |  |  |  |  |  |
|  |  | **Missing** | 1 | 11 |  |  |  |  |  |  |  |  |  |
|  |  | **TT** | 0 | 81 | 81.4 |  |  |  | 77.2 | 71.5 | 83.4 | - |  |
|  |  | **TC** | 1 | 114 | 113.2 |  |  |  | 78.5 | 73.6 | 83.8 | 1.7 |  |
|  |  | **CC** | 2 | 39 | 39.4 | 0.01 | 0.92 |  | 83.4 | 74.5 | 93.3 | 8.0 | 0.53 |
|  |  |  |  |  |  |  |  |  |  |  |  |  |  |
| ***TDG*** | **rs4135094**^d^ |  |  |  |  |  |  |  |  |  |  |  |  |
|  |  | **Missing** | 0 | 2 |  |  |  |  |  |  |  |  |  |
|  |  | **TT** | 0 | 106 | 106.7 |  |  |  | 77.5 | 73.8 | 81.3 | - |  |
|  |  | **TC** | 1 | 35 | 33.7 |  |  |  | 89.8 | 79.9 | 100.9 | 15.9 |  |
|  |  | **CC** | 2 | 2 | 2.7 | 0.22 | 0.64 |  | 97.2 | 59.7 | 158.3 | 25.5 | 0.05 |
|  |  | **TC+CC** |  | 37 |  |  |  |  | 90.2 | 80.5 | 100.9 | 16.4 | 0.02 |
|  |  |  |  |  |  |  |  |  |  |  |  |  |  |
| ***TDG*** | **rs167715**^d^ |  |  |  |  |  |  |  |  |  |  |  |  |
|  |  | **Missing** | 0 | 1 |  |  |  |  |  |  |  |  |  |
|  |  | **TT** | 0 | 192 | 188.6 |  |  |  | 80.2 | 76.3 | 84.4 | - |  |
|  |  | **TC** | 1 | 45 | 51.9 |  |  |  | 77.1 | 69.5 | 85.5 | -3.9 |  |
|  |  | **CC** | 2 | 7 | 3.6 | 4.28 | 0.04 |  | 68.8 | 52.9 | 89.4 | -14.3 | 0.44 |
|  |  | **TC+CC** |  | 52 |  |  |  |  | 75.9 | 68.9 | 83.6 | -5.4 | 0.32 |
|  |  |  |  |  |  |  |  |  |  |  |  |  |  |
| ***UNG*** | **rs3219245**^d^ |  |  |  |  |  |  |  |  |  |  |  |  |
|  |  | **Missing** | 0 | 4 |  |  |  |  |  |  |  |  |  |
|  |  | **GG** | 0 | 201 | 199.0 |  |  |  | 79.4 | 75.6 | 83.4 | - |  |
|  |  | **GT** | 1 | 36 | 40.0 |  |  |  | 78.5 | 69.9 | 88.2 | -1.1 |  |
|  |  | **TT** | 2 | 4 | 2.0 | 2.39 | 0.12 |  | 74.1 | 52.3 | 105.0 | -6.6 | 0.92 |
|  |  | **GT+TT** |  | 40 |  |  |  |  | 78.1 | 70.0 | 87.1 | -1.6 | 0.79 |
|  |  |  |  |  |  |  |  |  |  |  |  |  |  |
| ***UNG*** | **rs246079** |  |  |  |  |  |  |  |  |  |  |  |  |
|  |  | **Missing** | 1 | 2 |  |  |  |  |  |  |  |  |  |
|  |  | **AA** | 0 | 83 | 81.8 |  |  |  | 80.7 | 74.8 | 87.1 | - |  |
|  |  | **AG** | 1 | 116 | 118.4 |  |  |  | 78.5 | 73.6 | 83.7 | -2.7 |  |
|  |  | **GG** | 2 | 44 | 42.8 | 0.10 | 0.75 |  | 79.6 | 71.7 | 88.4 | -1.4 | 0.86 |
|  |  |  |  |  |  |  |  |  |  |  |  |  |  |
| ***XRCC1*** | **rs939461**^d^ |  |  |  |  |  |  |  |  |  |  |  |  |
|  |  | **Missing** | 0 | 1 |  |  |  |  |  |  |  |  |  |
|  |  | **AA** | 0 | 200 | 200.2 |  |  |  | 78.4 | 74.7 | 82.4 | - |  |
|  |  | **AC** | 1 | 42 | 41.7 |  |  |  | 82.9 | 74.5 | 92.3 | 5.7 |  |
|  |  | **CC** | 2 | 2 | 2.2 | 0.02 | 0.90 |  | 84.4 | 51.7 | 137.8 | 7.6 | 0.63 |
|  |  | **AC+CC** |  | 44 |  |  |  |  | 83.0 | 74.7 | 92.1 | 5.8 | 0.34 |
|  |  |  |  |  |  |  |  |  |  |  |  |  |  |
| ***XRCC1*** | **rs3213247**^d^ |  |  |  |  |  |  |  |  |  |  |  |  |
|  |  | **Missing** | 0 | 2 |  |  |  |  |  |  |  |  |  |
|  |  | **GG** | 0 | 213 | 213.9 |  |  |  | 78.9 | 75.2 | 82.7 | - |  |
|  |  | **GT** | 1 | 30 | 28.1 |  |  |  | 82.4 | 72.6 | 93.6 | 4.5 | 0.53 |
|  |  | **TT** | 2 | 0 | 0.9 | 1.05 | 0.31 |  |  |  |  |  |  |
|  |  | **GT+TT** |  | 30 |  |  |  |  | 82.4 | 72.6 | 93.6 | 4.5 | 0.43 |
|  |  |  |  |  |  |  |  |  |  |  |  |  |  |
| ***XRCC1*** | **rs939460** |  |  |  |  |  |  |  |  |  |  |  |  |
|  |  | **Missing** | 0 | 5 |  |  |  |  |  |  |  |  |  |
|  |  | **GG** | 0 | 168 | 165.8 |  |  |  | 82.1 | 77.9 | 86.7 | - |  |
|  |  | **GA** | 1 | 63 | 67.3 |  |  |  | 73.7 | 67.5 | 80.4 | -10.3 |  |
|  |  | **AA** | 2 | 9 | 6.8 | 0.99 | 0.32 |  | 70.6 | 55.7 | 89.3 | -14.1 | 0.07 |
|  |  | **GA+AA** |  | 72 |  |  |  |  | 73.3 | 67.6 | 79.5 | -10.8 | 0.02 |
|  |  |  |  |  |  |  |  |  |  |  |  |  |  |
| ***XRCC1*** | **rs25487** |  |  |  |  |  |  |  |  |  |  |  |  |
|  |  | **Missing** | 1 | 1 |  |  |  |  |  |  |  |  |  |
|  |  | **GG** | 0 | 91 | 91.6 |  |  |  | 77.4 | 72.0 | 83.3 | - |  |
|  |  | **GA** | 1 | 117 | 115.8 |  |  |  | 79.0 | 74.1 | 84.3 | 2.0 |  |
|  |  | **AA** | 2 | 36 | 36.6 | 0.03 | 0.87 |  | 84.8 | 75.4 | 95.2 | 9.5 | 0.43 |
|  |  |  |  |  |  |  |  |  |  |  |  |  |  |
| ***XRCC1*** | **rs25489**^d^ |  |  |  |  |  |  |  |  |  |  |  |  |
|  |  | **Missing** | 0 | 0 |  |  |  |  |  |  |  |  |  |
|  |  | **GG** | 0 | 222 | 220.6 |  |  |  | 81.9 | 78.2 | 85.8 | - |  |
|  |  | **GA** | 1 | 21 | 23.7 |  |  |  | 81.6 | 69.5 | 95.7 | -0.4 |  |
|  |  | **AA** | 2 | 2 | 0.6 | 3.23 | 0.07 |  | 75.2 | 46.6 | 121.5 | -8.1 | 0.93 |
|  |  | **GA+AA** |  | 23 |  |  |  |  | 80.9 | 69.6 | 94.0 | -1.2 | 0.98 |
|  |  |  |  |  |  |  |  |  |  |  |  |  |  |
| ***XRCC1*** | **rs1001581** |  |  |  |  |  |  |  |  |  |  |  |  |
|  |  | **Missing** | 1 | 3 |  |  |  |  |  |  |  |  |  |
|  |  | **CC** | 0 | 85 | 86.3 |  |  |  | 78.3 | 72.7 | 84.4 | - |  |
|  |  | **CT** | 1 | 119 | 116.4 |  |  |  | 77.0 | 72.3 | 82.1 | -1.7 |  |
|  |  | **TT** | 2 | 38 | 39.3 | 0.12 | 0.73 |  | 87.8 | 78.4 | 98.3 | 12.1 | 0.14 |
|  |  |  |  |  |  |  |  |  |  |  |  |  |  |
| ***XRCC1*** | **rs2307191** |  |  |  |  |  |  |  |  |  |  |  |  |
|  |  | **Missing** | 0 | 6 |  |  |  |  |  |  |  |  |  |
|  |  | **CC** | 0 | 239 |  |  |  |  | 79.2 | 75.7 | 82.9 |  | NA |
|  |  |  |  |  |  |  |  |  |  |  |  |  |  |
| ***XRCC1*** | **rs3213403**^d^ |  |  |  |  |  |  |  |  |  |  |  |  |
|  |  | **Missing** | 0 | 1 |  |  |  |  |  |  |  |  |  |
|  |  | **AA** | 0 | 21 | 24.0 |  |  |  | 79.2 | 75.5 | 83.1 | - |  |
|  |  | **AG** | 1 | 28 | 22.0 |  |  |  | 78.4 | 68.6 | 89.4 | -1.0 |  |
|  |  | **GG** | 2 | 2 | 5.0 | 3.86 | 0.05 |  | 99.8 | 60.8 | 163.7 | 26.1 | 0.65 |
|  |  | **AG+GG** |  | 30 |  |  |  |  | 79.6 | 70.1 | 90.5 | 0.6 | 0.94 |
|  |  |  |  |  |  |  |  |  |  |  |  |  |  |
| ***XRCC1*** | **rs915927** |  |  |  |  |  |  |  |  |  |  |  |  |
|  |  | **Missing** | 1 | 11 |  |  |  |  |  |  |  |  |  |
|  |  | **AA** | 0 | 81 | 82.0 |  |  |  | 81.5 | 75.5 | 88.0 | - |  |
|  |  | **AG** | 1 | 115 | 113.0 |  |  |  | 80.7 | 75.7 | 86.0 | -1.0 |  |
|  |  | **GG** | 2 | 38 | 39.0 | 0.07 | 0.79 |  | 72.1 | 64.5 | 80.6 | -11.5 | 0.17 |
|  |  |  |  |  |  |  |  |  |  |  |  |  |  |
| ***XRCC1*** | **rs3213255** |  |  |  |  |  |  |  |  |  |  |  |  |
|  |  | **Missing** | 1 | 12 |  |  |  |  |  |  |  |  |  |
|  |  | **TT** | 0 | 93 | 93.4 |  |  |  | 81.1 | 75.5 | 87.2 | - |  |
|  |  | **TC** | 1 | 109 | 108.3 |  |  |  | 79.6 | 74.4 | 85.0 | -1.9 |  |
|  |  | **CC** | 2 | 31 | 31.4 | 0.01 | 0.92 |  | 70.9 | 62.6 | 80.4 | -12.6 | 0.18 |
|  |  |  |  |  |  |  |  |  |  |  |  |  |  |
| ***XRCC1*** | **rs25496** |  |  |  |  |  |  |  |  |  |  |  |  |
|  |  | **Missing** | 0 | 4 |  |  |  |  |  |  |  |  |  |
|  |  | **TT** | 0 | 241 |  |  |  |  | 79.0 | 75.6 | 82.6 |  | NA |

Abbreviations: CL, confidence limit; Diff., difference; HWE, Hardy-Weinberg Equilibrium; LL, lower limit; MAP, Markers of Adenomatous Polyps; Prop. diff., proportional difference; SNP, single nucleotide polymorphism; UL, upper limit.

^a^ Geometric means, 95% confidence limits, and *P*-values from general linear models, adjusted for sex (male/female) and body mass index (continuous).

^b^ Based on minor allele frequency in the European population in the 1000 Genomes Project Phase 3 (ensembl.org OR ncbi.nlm.nih.gov/projects/SNP).

^c^ Proportional difference, in percent, between mean plasma F_2_-isoprostanes concentration among those with a variant genotype relative to those with the common homozygous genotype; i.e.: [(variant mean - common mean) / common mean] x 100%.

^d^ Heterozygous and/or variant homozygous genotypes with ≤ 10 participants were combined.

**Supplementary Table S7.** Mean^a^ plasma F_2_-isoprostanes concentrations, according to tertiles of an oxidative balance score (OBS)^b^, in the pooled MAP I and MAP II cross-sectional studies (*n* = 245).

| **Model/OBS tertiles** | **OBS tertile medians** | **Plasma FiP, pg/mL** | | | | |
| --- | --- | --- | --- | --- | --- | --- |
|  |  | *n* | Means | (95% CI) | Prop. diff.^c^ (%) | *p* |
| Crude^d^ |  |  |  |  |  |  |
| OBS tertiles |  |  |  |  |  |  |
| 1 | -2.74 | 79 | 99.0 | (91.3, 107.4) | Ref. |  |
| 2 | -0.48 | 86 | 81.1 | (75.1, 87.7) | -18.1 |  |
| 3 | 1.27 | 80 | 64.8 | (59.8, 70.3) | -34.5 | *<0.001* |
|  |  |  |  |  |  |  |
| Multivariable-adjusted^e^ | |  |  |  |  |  |
| *OBS tertiles* |  |  |  |  |  |  |
| 1 | -2.74 | 79 | 101.7 | (93, 111.2) | Ref. |  |
| 2 | -0.48 | 86 | 85.0 | (77.9, 92.8) | -16.4 |  |
| 3 | 1.27 | 80 | 69.2 | (63.4, 75.4) | -32.0 | *<0.001* |

Abbreviations: CI, confidence interval; FiP, F_2_-isoprostanes; MAP, Markers of Adenomatous Polyps; OBS, oxidative balance score; Prop. diff., proportional difference; Ref., reference.

^a^ Geometric means, 95% confidence intervals, and *p*-values from general linear models.

^b^ Oxidative balance score a composite of 17 weighted anti- and pro-oxidant dietary and lifestyle exposures (see text); a higher score represents a higher balance of anti- relative to pro-oxidant exposures.

^c^ Proportional difference calculated as (comparison group mean - reference group mean) / (reference group mean) x 100%.

^d^ No covariates in the model.

^e^ Adjusted for total energy intake, sex, education (less than high school, high school degree, college graduate or higher), current hormone replacement therapy use (among women), aspirin and/or other nonsteroidal anti-inflammatory drug use (≥ 1/wk or < 1/wk).

**Supplementary References**

Ba, X., Aguilera-Aguirre, L., Rashid, Q.T., Bacsi, A., Radak, Z., Sur, S., et al. (2014). The role of 8-oxoguanine DNA glycosylase-1 in inflammation. *Int J Mol Sci* 15(9)**,** 16975-16997. doi: 10.3390/ijms150916975.

Duell, E.J., Wiencke, J.K., Cheng, T.J., Varkonyi, A., Zuo, Z.F., Ashok, T.D., et al. (2000). Polymorphisms in the DNA repair genes XRCC1 and ERCC2 and biomarkers of DNA damage in human blood mononuclear cells. *Carcinogenesis* 21(5)**,** 965-971. doi: 10.1093/carcin/21.5.965.

Harrington, J.J., and Lieber, M.R. (1994). The characterization of a mammalian DNA structure‐specific endonuclease. *The EMBO journal* 13(5)**,** 1235-1246.

He, Y.F., Li, B.Z., Li, Z., Liu, P., Wang, Y., Tang, Q., et al. (2011). Tet-mediated formation of 5-carboxylcytosine and its excision by TDG in mammalian DNA. *Science* 333(6047)**,** 1303-1307. doi: 10.1126/science.1210944.

Henderson, C.J., McLaren, A.W., Moffat, G.J., Bacon, E.J., and Wolf, C.R. (1998). Pi-class glutathione S-transferase: regulation and function. *Chem Biol Interact* 111-112**,** 69-82. doi: 10.1016/s0009-2797(97)00176-2.

Kucherlapati, M., Yang, K., Kuraguchi, M., Zhao, J., Lia, M., Heyer, J., et al. (2002). Haploinsufficiency of Flap endonuclease (Fen1) leads to rapid tumor progression. *Proceedings of the National Academy of Sciences* 99(15)**,** 9924-9929.

Moyer, A.M., Salavaggione, O.E., Wu, T.Y., Moon, I., Eckloff, B.W., Hildebrandt, M.A., et al. (2008). Glutathione s-transferase p1: gene sequence variation and functional genomic studies. *Cancer Res* 68(12)**,** 4791-4801. doi: 10.1158/0008-5472.Can-07-6724.

Nielsen, M., Morreau, H., Vasen, H.F., and Hes, F.J. (2011). MUTYH-associated polyposis (MAP). *Crit Rev Oncol Hematol* 79(1)**,** 1-16. doi: 10.1016/j.critrevonc.2010.05.011.

Sampson, J.R., Jones, S., Dolwani, S., and Cheadle, J.P. (2005). MutYH (MYH) and colorectal cancer. *Biochem Soc Trans* 33(Pt 4)**,** 679-683. doi: 10.1042/bst0330679.

Thompson, L.H., and West, M.G. (2000). XRCC1 keeps DNA from getting stranded. *Mutat Res* 459(1)**,** 1-18. doi: 10.1016/s0921-8777(99)00058-0.

Wu, X., and Zhang, Y. (2017). TET-mediated active DNA demethylation: mechanism, function and beyond. *Nat Rev Genet* 18(9)**,** 517-534. doi: 10.1038/nrg.2017.33.
